# Marked unergatives: Syntactic ergativity and nominalizations

**DOI:** 10.1007/s11049-025-09672-6

**Published:** 2025-07-17

**Authors:** Jens Hopperdietzel, Artemis Alexiadou

**Affiliations:** 1 Department of German Language and Literature I, University of Cologne, Albertus Magnus Platz, 50923 Cologne, Germany; 2 Department of Linguistics and English Language, University of Manchester, Oxford Road, Manchester, M139PL UK; 3 Leibniz-Centre General Linguistics (ZAS), Pariser Straße 1, 10719 Berlin, Germany; 4 Department of German Studies and Linguistics, Humboldt-University of Berlin, Unter den Linden 6, 10117 Berlin, Germany

**Keywords:** Nominalizations, Argument structure, (in)alienability, Ergativity, Subject clitics, Polynesian

## Abstract

Samoan deverbal nominalizations show a crosslinguistically rare tripartite-inactive alignment where unaccusative, unergative, and transitive subjects are distinguished by inalienable genitive, alienable genitive, and ergative case, respectively, with objects being marked like unaccusative subjects (Mosel 1992). In addition, subject clitics exhibit a marked unergative alignment, where only unergative subject clitics are distinctly marked by alienable genitive case, whereas all other arguments receive inalienable genitive case. In this study, we demonstrate that these alignments follow naturally from a language-specific combination of independently established phenomena, including (i) prepositional ergativity (Polinsky 2016), (ii) split (in)alienability (Myler 2016; Alexiadou 2003), (iii) split-intransitivity, (iv) the unaccusative restriction on nominalizations (Imanishi 2014; Alexiadou 2001), and (v) a nonuniform nature of clitic pronouns (Bleam 2000), and therefore provides novel evidence for each of these phenomena. Comparing the distribution of ergative case in nominalizations crosslinguistically, we argue that the source of ergativity varies across languages and suggest that the split between syntactic and morphological ergativity cannot be reduced to a category-split of ergative subjects.

## Introduction

Deverbal nominalizations as mixed extended projections show properties traditionally related to both the nominal and the verbal domain, such as argument and event structure, plural marking, adjectival and adverbial modification, etc. (Alexiadou and Borer 2020; Borer 2014; Harley 2009; Alexiadou et al. 2007; Alexiadou 2001; Grimshaw 1990 and many others); it has also been acknowledged that different types of nominalizations vary in the exact distribution of such properties: so-called complex event nominalizations entail the event and argument structure of the nominalized verbal predicate whereas result nouns do not (Grimshaw 1990 and much subsequent work). Complex event nominalizations have thereby been commonly described to exhibit specific restrictions on the syntactic realization of their arguments: On the one hand, nominal arguments are typically marked by nominal genitive/possessive case instead of verbal cases like nominative (1)a or accusative (1)b, as illustrated by Greek below.^1^

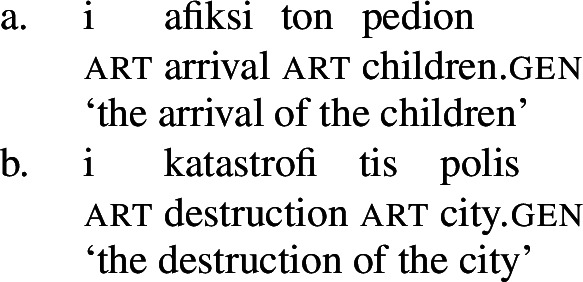

On the other hand, certain nominalizations typically require the external argument of transitive verbs, i.e., the agent, to be introduced by an (optional) prepositional *by*-phrase, similar to passive constructions (2), instead of a DP (Bruening 2013; Grimshaw 1990). As a result, even accusative languages like Greek show an ergative alignment in the nominal domain (Alexiadou 2017, 2001).

(2)
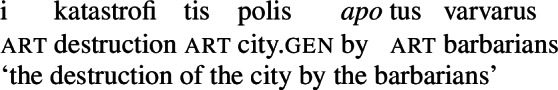

Based on this restriction, nominalizations have been argued to be subject to an unaccusativity requirement (3), according to which only unaccusative structures can be nominalized (Imanishi 2020; Alexiadou 2001).

(3)
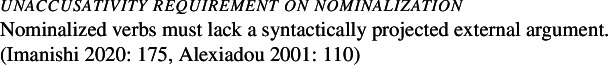

While most studies focus on the subject of transitive verbs, the unaccusative requirement also affects the realization of the external argument of nominalized unergative verbs, although their status as complex event nominals has been contended (Grimshaw 1990, cf. Borer 2013). In Greek, such arguments lose their agentive properties in nominalizations and are instead introduced as a possessor, as indicated by the ungrammaticality of agent-oriented modifiers in (4) (Alexiadou 2001).

(4)
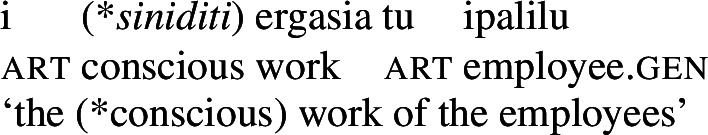

Although the initial motivation for the unaccusative requirement on nominalizations came from Indo-European languages like English, Catalan, and Greek (see Alexiadou 2001 and references therein), parallel restrictions have been described for typologically unrelated languages like Kaqchikel (Mayan; Imanishi 2020, 2014) and Mẽbêngôkre (Jê; Salanova 2007), suggesting a more universal constraint on argument structure in complex event nominals. Yet, crosslinguistic data on the status of external arguments in nominalizations is still rare, especially from nonaccusative languages. Moreover, recent studies challenge the universal character of the unaccusative restriction based on potential counterexamples from a diverse set of languages (Šereikaitė 2023 on Lithuanian, Imanishi 2020 on Chuj and Ch’ol (Mayan), Smirnova and Jackendoff 2017 on Russian, Massam 2000 on Niuean (Polynesian)), suggesting a parametrized view on nominalizations (Imanishi 2020, cf. Alexiadou to appear).

In this study, we present novel data from Samoan (Polynesian) which provides independent evidence for the unaccusative requirement from a combination of verbal and nominal case alignments in complex event nominalizations. While Samoan exhibits an ergative alignment in the verbal/clausal domain and a split-genitive alignment in the nominal domain, nominalizations show a tripartite-inactive alignment on regular DP arguments that distinguishes unaccusative, unergative, and transitive subjects (Mosel 1992; Mosel and Hovdhaugen 1992, cf. Chung 1973): Transitive subjects maintain their ergative marking (5)c, whereas intransitive unergative (5)b and unaccusative subjects (5)a are marked by alienable and inalienable genitive case, respectively.

(5)
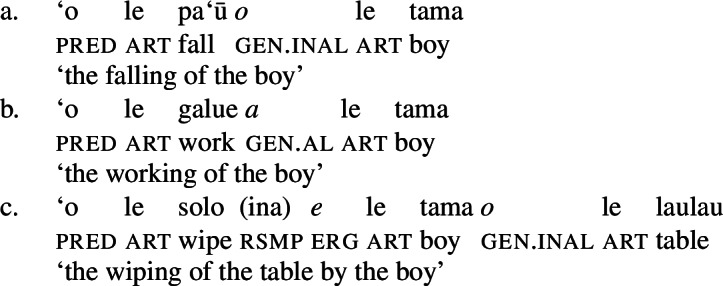

Based on their syntactic and semantic properties, we demonstrate that this typologically rare alignment follows naturally from two independent properties of the Samoan grammar that combine with the more general unaccusative requirement in complex event nominalizations: Firstly, the prepositional nature of transitive subjects in a syntactically ergative language like Samoan (Hopperdietzel 2020; Polinsky 2016; Mosel 1985), which makes them similar to passive *by-*phrases in accusative languages and allows them to escape the unaccusative requirement on nominalizations. Secondly, inherent alienable genitive case on unergative subjects indicates that only unergative subjects must be based-generated outside of the verbal domain, namely as possessors in the nominal domain, as only they fail to satisfy the unaccusative requirement (cf. Tyler 2021; Myler 2016; Alexiadou 2003).

(6)
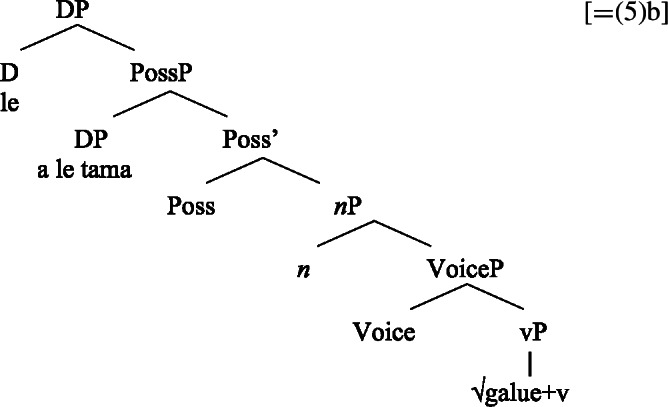

Additional support for the special status of unergative subjects comes from subject clitics which in verbal clauses exhibit a neutralized alignment with the three types of subject being marked by absolutive case in the absence of ergative marking (Mosel and Hovdhaugen 1992). In complex event nominalizations, however, unergative subjects are distinctly marked by alienable genitive case (7)b, whereas transitive subjects (7)c pattern with unaccusative subjects being marked by inalienable genitive case (7)a (Mosel 1992).

(7)
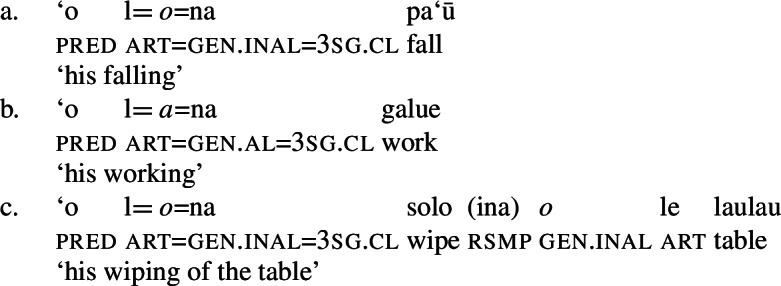

Based on the presence of the prepositional resumptive pronoun *ina* only in transitive contexts, we develop a nonuniform analysis of subject clitics in Samoan (cf. Bleam 2000): While intransitive subject clitics receive both their case and thematic role in their original argument position before moving to a preverbal position (cf. Uriagereka 1995), transitive subjects are base-generated in a nonthematic position (cf. Sportiche 1996), receiving the unmarked case of the respective domain (Baker 2015), with their thematic role assigned via resumption. As a result, in nominalizations, unergative subject clitics are both case and thematically licensed in the nominal domain, indicated by alienable genitive case and interpretative shifts.

The marked unergative alignment in Samoan nominalizations therefore supports the absence of external arguments in complex event nominalizations and provides novel crosslinguistic evidence for the unaccusative restriction on nominalizations from an ergative language. Crucially, marked unergatives make further predictions about the source of syntactic ergativity in the language, which we argue, is tied to the prepositional nature of the ergative subject in Samoan. Contrasting the Samoan data with nominalizations from the morphologically ergative language Niuean, which does not display syntactic ergativity and rejects ergative marking in complex event nominalizations (Massam 2020, 2000), our findings support the view that ergative subjects in morphologically and syntactically ergative languages differ regarding their lexical category, i.e., DP vs. PP (Polinsky 2016). However, given that ergative subjects have been argued to violate the unaccusative requirement on nominalizations, e.g., in the Mayan language Kaqchikel (Imanishi 2020, 2014, cf. Burukina 2023), we suggest that syntactic ergativity is not a crosslinguistically uniform phenomenon related to prepositional ergativity (*pace* Polinsky 2016, cf. Coon et al. 2014). Consequently, the distribution of ergative case in nominalization may be used as a diagnostic for the categorial status of subjects of transitive verbs in future research.

The paper is structured as follows: In Sect. 2, we start our investigation with an overview of the case alignments in Samoan, showing that it exhibits an ergative alignment in the clausal domain and a split-genitive alignment in the nominal domain that is sensitive to (in)alienability. We then turn to the tripartite-inactive alignment found in Samoan complex event nominalizations, in which unaccusative, unergative, and transitive subjects are marked by distinct morphological cases. After demonstrating that Samoan complex event nominalizations embed a VoiceP under the nominalizer *n*, we explain this alignment by the unaccusative restriction on nominalization, according to which *n* selects for unaccusative complements. While unergative subjects are forced to be introduced in the nominal domain, we argue that ergative subjects obey this requirement due to their prepositional nature in a syntactically ergative language like Samoan. In Sect. 3, we apply our analysis to the marked unergative alignment of subject clitics. Developing a nonuniform analysis of subject clitics in Samoan, we propose that only transitive subject clitics are base-generated in T, receiving default case, whereas intransitive subject clitics are merged in the original argument position before moving to T. Due to the unaccusative restriction and the absence of prepositional resumption, unergative clitics must be merged in the nominal domain where they receive both inherent alienable genitive case and the possessor role in Spec, PossP. In Sect. 4, we discuss how our findings support a prepositional account of syntactic ergativity in Samoan and how nominalizations can inform the analysis of source of (syntactic) ergativity crosslinguistically. Section 5 concludes.

## Tripartite-inactive case

Although argument structure properties of deverbal nominalizations have received much attention in the typological and formal literature (e.g., Alexiadou and Borer 2020; Koptjevskaja-Tamm 1993), the morphosyntactic relations between the nominalized verb and its arguments have been investigated primarily from the perspective of better-studied accusative languages. In such languages, arguments in deverbal nominalizations typically exhibit an ergative alignment where subjects of transitive verbs are distinctly marked from intransitive subjects and objects (1)/(2) (Alexiadou 2017, 2001). Based on this observation, certain nominalizations in these languages have been argued to be subject to an unaccusative requirement that prevents the syntactic projection of an external argument in Spec, VoiceP, making nominalizations similar to passives (Imanishi 2020; Bruening 2013; Alexiadou 2001). Despite its strong crosslinguistic prediction, data from a typologically more diverse set of languages, especially languages that do not exhibit an accusative alignment, is rarely discussed (but see Burukina 2023, 2021; Imanishi 2020, 2014 on various Mayan languages, Polinsky 2016 on Tongan, Salanova 2007 on Mẽbêngôkre, Massam 2000 on Niuean).

In this section, we will contribute to this discussion data from the Polynesian language Samoan, which combines an ergative alignment in the clausal domain with a split genitive alignment sensitive to (in)alienability in the nominal domain to a crosslinguistically rare tripartite-inactive case alignment in deverbal nominalizations (e.g., not discussed by Comrie 2013), where unaccusative, unergative, and transitive subjects are distinguished by their case marking. Crucially, Samoan differs from Mayan languages in that its ergative is not syncretic with the genitive (Coon 2013), providing more direct morphological access to the syntactic status of the respective arguments. Adopting a prepositional analysis of syntactic ergativity in Samoan (Hopperdietzel 2020; Polinsky 2016; Mosel 1985, *pace* Collins 2021; Tollan 2018), we then argue that the tripartite-inactive alignment naturally follows from the unaccusative restriction on Voice-under-*n* in complex event nominalizations in the presence of an inherent alienable genitive case assigned by Poss (cf. Tyler 2021 on Mississippi Choctaw, Alexiadou 2001). The tripartite-inactive case alignment in Samoan nominalizations therefore provides independent support for the unaccusative restriction on nominalizations, a syntactic approach on (in)alienability, and a prepositional analysis of syntactic ergativity in the language (see Sect. 4.1 for a more detailed discussion).

### Typological and methodological background

Samoan is a Polynesian language of the Oceanic subbranch of the Austronesian language family and spoken by roughly 510 000 speakers of which half of the population lives on the islands of the Samoan archipelago, i.e., the Independent State of Samoa and American Samoa (Lewis et al. 2015). In addition, larger communities reside in Australia, New Zealand, and the USA, especially in Hawai*`*i and the West Coast. The language is primarily VSO, with other word orders likely sensitive to information structure, and exhibits preverbal TMA marking and postverbal adverbial modifiers, as illustrated below (see Mosel and Hovdhaugen 1992 for a reference grammar).

(8)
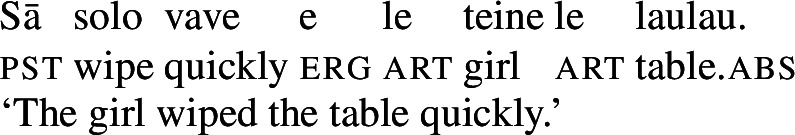

The structure of the DP is parallel to the clausal structure with prenominal case-marking and articles and postnominal modifiers and possessor arguments.

(9)
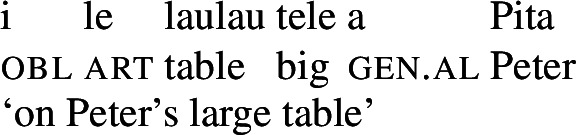

To account for the verb-initial syntax, we adopt an analysis based on phrasal VP movement to the Spec, TP and additional T to C movement (Collins 2017, cf. Medeiros 2013; Massam 2001 on remnant movement in Hawai’ian and Niuean, also Middleton 2021; Otsuka 2005 in T-to-C movement in Polynesian languages), combined with distributed deletion of nonverbal (phasal) constituents, including DPs, PPs, and CPs, in the higher copy (van Urk 2022, cf. Clemens 2014).^2^ This is illustrated by the structure in (10) for the sentence in (8), without the postverbal adverbial *vave* ‘quickly’.

(10)
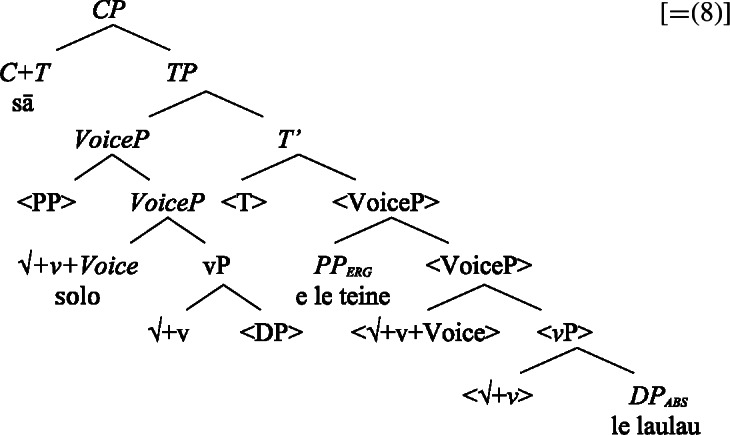

Following Kahnemuyipour and Massam (2006 on Niuean) and Pearce (2005 on Maori), we assume a parallel account for noun-initial syntax with the NP movement to Spec, DP, stranding of phasal arguments in postnominal position, and subsequent D-to-K movement. As linearization is not crucial to our study here, we will present hierarchical structures prior to movement for presentational convenience.

Most data presented in this paper comes from elicitation sessions with five native speakers of Samoan, of which two speakers (one male, one female; born and raised in Western Samoa) live in the United States and three speakers on the island of Upolu in the Independent State of Samoa (two female, one male). Dialectal and interspeaker variation that occurred during the elicitation sessions are mentioned, where relevant. Samoan exhibits two registers, a more formal and a more colloquial one, called *t*- and *k-style*. All examples are presented in the *t*-style, which was voluntarily offered by our consultants. Apart from phonological differences, e.g., /t/ to /k/, while registers only have been described to exhibit a minor impact on the morphosyntax, case marking may be affected (Collins 2014, cf. Mayer 2001; Ochs 1982; Duranti 1981). The data was elicited in on- and offline meetings between 2021 and 2023 via judgment tasks based on manipulated examples, partly adapted from existing sources on Samoan nominalizations (Mosel 1992; Mosel and Hovdhaugen 1992; Chung 1973), as well as translation tasks based on English stimuli. While all examples and stimuli were tested in the context of full clauses, we decided to present only the relevant part, i.e., the nominalization itself for presentational clarity.

### Samoan case

In the following, we focus on Samoan case alignment in the verbal and the nominal domain before we turn to the combination of both alignments in deverbal nominalizations. In particular, we adopt a prepositional analysis of syntactic ergativity and a structural analysis of the split-genitive alignment sensitive to (in)alienability, which will be crucial for the analysis of the observed tripartite-inactive in deverbal nominalizations.

#### The clausal domain: Ergative/absolutive alignment

In the clausal domain, Samoan exhibits an ergative/absolutive case alignment (Mosel and Hovdhaugen 1992).^3^ The transitive subject is marked by the ergative marker *e* (11),^4^ whereas intransitive subjects and objects are marked by tonal absolutive case, which is realized by a pitch rise on the preceding syllable (Yu 2021).^5^

(11)
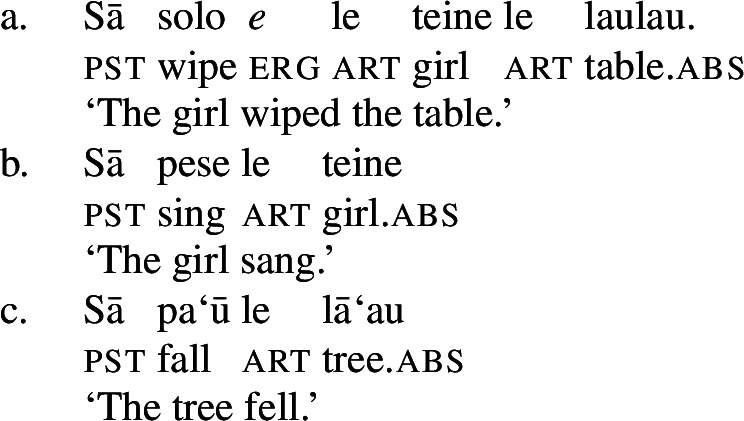

Notably, Samoan shows properties of syntactic ergativity as ergative arguments reject A’-movement to a clause-initial position (Muāgututi’a 2018; Polinsky 2016; Mosel and Hovdhaugen 1992). In topicalizations, for example, absolutive arguments move to a clause-initial position without any additional material present (12)a (see Hohaus and Howell 2015; Potsdam and Polinsky 2011 for a discussion of *`o* in Samon and its cognates in other Polynesian languages). Topicalized ergative subjects however drop their ergative marking and the resumptive pronoun *ina* shows up in the original subject position (12)b (Hopperdietzel 2020; Cook 1988, also Ershova 2024).^6^

(12)
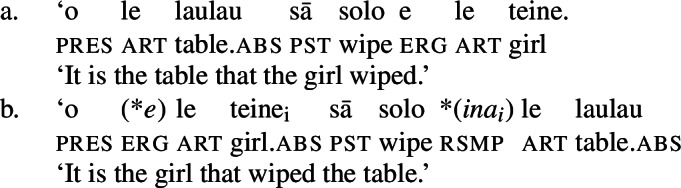

Ergative subjects thus resemble other oblique PPs in the language which drop their oblique marker *i* in topicalized position and require the presence of a resumptive pronoun, like *i ai*, in postverbal position.

(13)
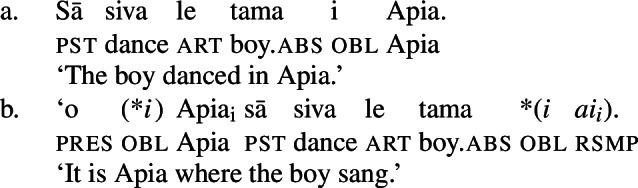

Adopting Polinsky’s (2016) prepositional analysis of syntactic ergativity, we take ergative and oblique marking to be inherently prepositional. In particular, we assume that ergative subjects (left) adjoin as (passive-like) prepositional adjuncts to VoiceP, saturating the agent role introduced by Voice (10) (cf. Mosel 1985 on the adjunct-like optionality of ergative arguments in isolated sentences). Since Samoan lacks both preposition stranding and pied-piping (13)b, prepositional constituents, including the ergative subject, are trapped in their base-generated position. The topicalization of prepositional arguments therefore requires resumption, in which the argument is base-generated as a DP argument in the left periphery and resumed by a prepositional anaphoric pro-form in its original VoiceP-internal position.^7^ This is illustrated for topicalized ergative subjects in (14).

(14)
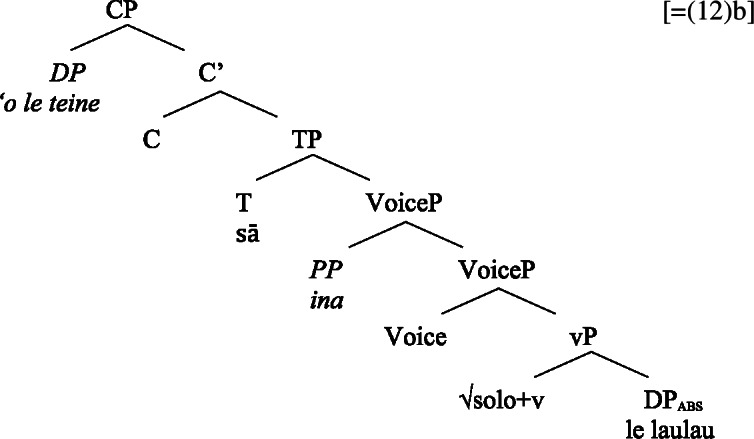

This analysis is supported by the morphological markup of the ergative resumptive pronoun, which can be decomposed into the general oblique marker *i* and the deictic demonstrative proform *nā* ‘that there’ (Mosel and Hovdhaugen 1992: 131f).^8^ As discussed in detail by Polinsky (2016) for Tongan, prepositional ergativity is expected to correlate with additional morphosyntactic phenomena, particularly anaphoric binding, raising, and control. Polinsky (2016) also offers tentative evidence that these patterns hold in Samoan (*pace* Chung 1978), though a systematic investigation lies beyond the scope of this paper: Firstly, Samoan lacks reflexive anaphoric pronouns (Mosel 1991). Secondly, potential (hyper-)raising structures seem to be only apparent, as ‘raising’ can target nonsubjects, including obliques (e.g., *masani-*raising), can (optionally) leave a pronominal copy in the embedded clause, and lacks case connectivity of the ‘raised’ argument (15) (cf. Chung 1978 for a discussion).^9^

(15)
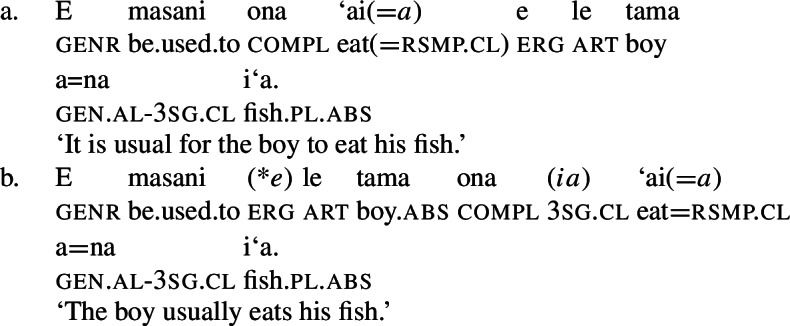

Finally, potential control structures show properties of nonobligatory control, as they allow binding by noncommanding antecedents (16) (Polinsky 2016), and optional overt pronouns in the subordinated clause.

(16)
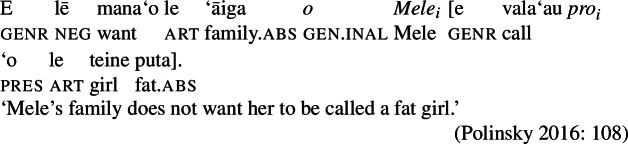

Note that the label transitive/ergative “subject” as used in this study is therefore somewhat misleading, since ergative PPs are more similar to oblique PPs, such as passive *by*-phrases in English, with the absolutive argument being the “true” subject of the clause (cf. Chung 1978; Hohepa 1969 on syncretism of ergative subjects and passive *by*-phrases in Western and Eastern Polynesian languages). Despite this complication, we continue to refer to ergative PPs as transitive subjects, following the tradition of Polynesian grammar description (but see Cook 1991; Mosel 1985 for a critical discussion of the notion of “subject” in Samoan). We further emphasize that our analysis of the ergative case marker as a preposition is specific to Samoan (and potentially to other Polynesian languages) and is not intended as a universal claim about ergativity across languages. In particular, we make no claims about the source of syntactic ergativity in unrelated language families, where similar surface patterns may arise from distinct underlying structures; for example, high absolutive syntax in Mayan languages (cf. Coon et al. 2014).

In contrast to inherent/prepositional ergative marking, we treat absolutive case as the unmarked case in the clausal domain (Baker 2015; McFadden 2004; Marantz 1991), though our analysis is fully compatible with traditional agree-based approaches. In line with a high-absolutive analysis of Samoan (Hopperdietzel 2020; Tollan 2018; Koopman 2012), we assume that absolutive case is assigned to the highest DP in the TP. Evidence for this assumption comes from the unavailability of absolutive case in nonfinite contexts in which T is absent. This is illustrated by bare nominalizations below, which lack both T and absolutive case (see Sect. 2.3.2 for a detailed discussion; *pace* Collins 2014).

(17)
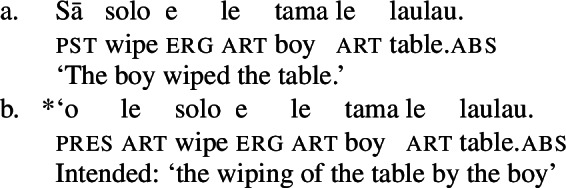

Absolutive case in Samoan is therefore equivalent to nominative case in accusative languages like English or Greek (ABS=NOM; Legate 2008), where nominative is assigned to internal arguments in passive constructions. If Samoan absolutive case were accusative case in transitive clauses (ABS=DEF), we would expect absolutive case to be available in nominalizations, especially in the presence of ergative marking on the transitive subject (but see FN15 for a more complex empirical picture). As regular DPs, absolutive arguments can freely undergo A’-movement to a clause-initial position in topicalization, as illustrated for (12)a below.

(18)
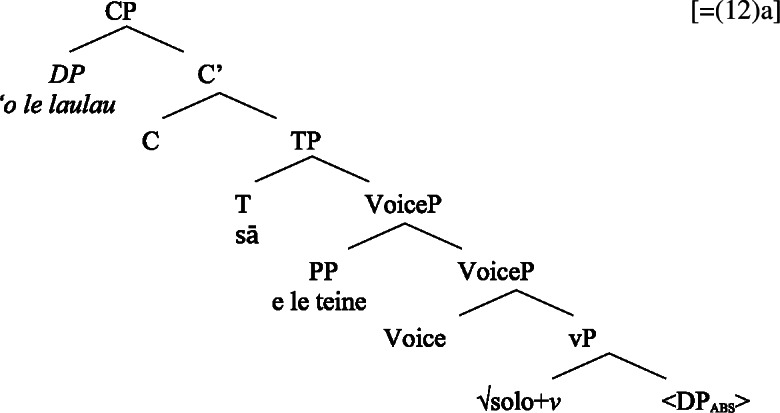

To summarize, Samoan exhibits an ergative alignment with ergative marking being an inherent prepositional case. Ergative subjects thus resemble passive *by*-phrases, suggesting that semantically transitive clauses are syntactically intransitive in the narrow sense (cf. Mosel 1985, see Otsuka 2011 for a similar intuition on Polynesian).^10^

#### The nominal domain: Split-genitive alignment

In the nominal domain, Samoan like other Polynesian languages distinguishes between alienable and inalienable possessors morphologically by distinct genitive cases on the possessor (Mayer 2001; Mosel and Hovdhaugen 1992: 282ff, cf. Clark 2000; Wilson 1982; Biggs 1969). On the one hand, inalienable possessors, including body parts (19)a, part–whole relationships (19)b, and characteristic properties (19)c, are marked by the case marker *o*.^11^

(19)

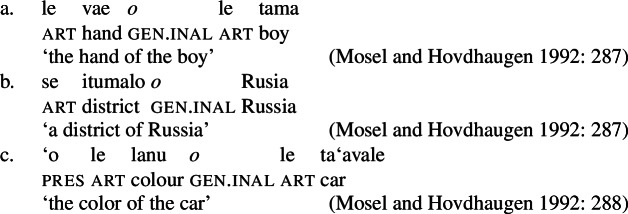

On the other hand, alienable possessors are marked by the genitive case marker *a*, including nonpermanent and initiated possession (20)a/b and transitory relationships (20)c.

(20)
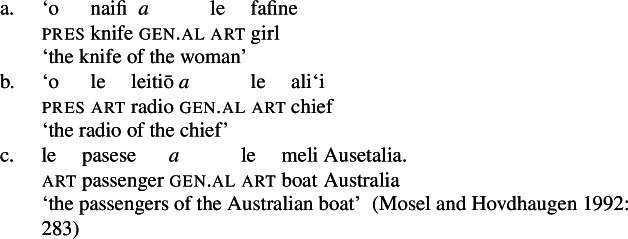

Some nouns can combine with both alienable and inalienable possessors, depending on the possessive relationship that holds between the two nouns.

(21)
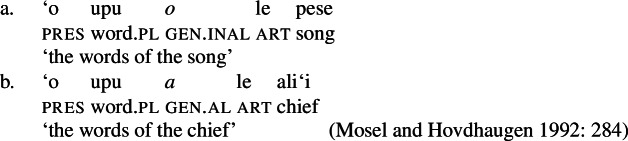

(22)
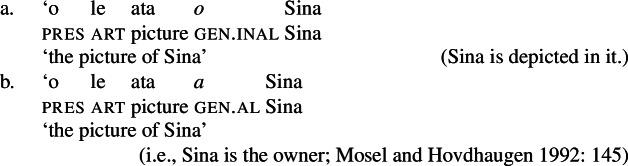

Notably, alienable and inalienable genitive marking can cooccur within a single noun phrase, suggesting independent morphosyntactic sources for inalienable and alienable genitive case (see also Ball 2009 on Tongan).^12^

(23)
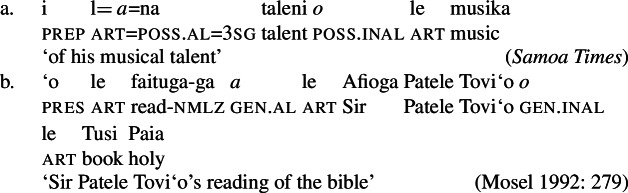

Adopting a structural analysis of (in)alienability (Armstrong 2024; Tyler 2021; Myler 2016; Alexiadou 2003, 2001; Szabolsci 1994 and references therein), we assume that inalienable possessors merge *n*P-internally as direct arguments of relational nouns. As such, they receive inalienable genitive case, which we take to be the unmarked case in the DP domain, equivalent to absolutive case in the verbal domain (cf. Baker 2015; see Sect. 4 for further parallels between absolutive and (inalienable) genitive case), as illustrated for example (19)a in (24) below.

(24)
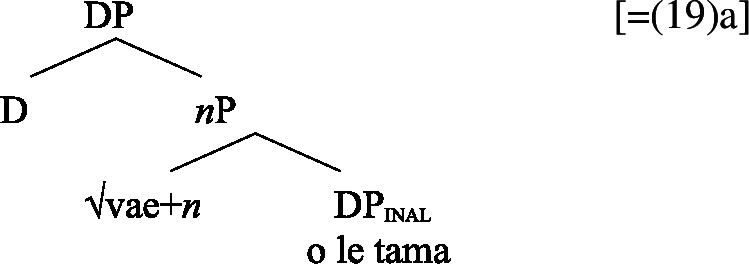

In contrast, alienable possessors are merged in the specifier of an additional functional head Poss, parallel to Voice in the verbal domain, which on the semantic level, introduces an abstract possessive relation between the two nouns (Barker 1995). While in languages like English or Greek, where inalienable and alienable possessors are marked alike, the structural distinction between alienable and inalienable possessors is not reflected by morphology, alienable genitive case is inherently assigned to *n*P-external argument DPs in Spec, PossP in languages like Samoan (cf. Tyler 2021 on inherent dative case on alienable possessors in Mississippi Choctaw).^13^

(25)
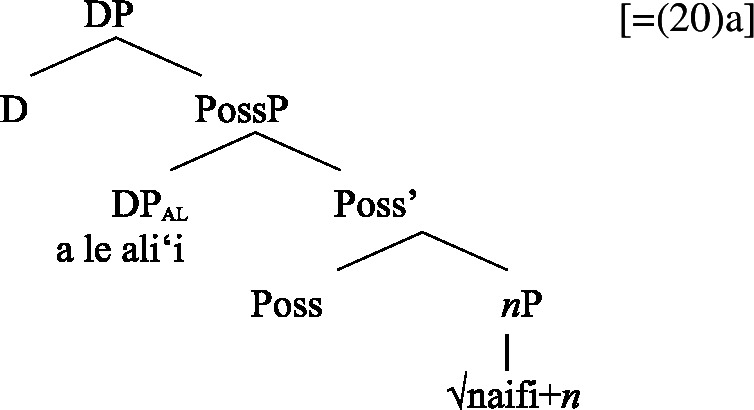

Alienable genitive case in Samoan is therefore an inherent case in the nominal domain, similar to morphological ergative case in languages like Basque, where *v*P-external arguments, i.e., subjects of unergatives and transitive verbs, are uniformly marked by ergative case (e.g., Holmer 1999).

#### Deverbal nominalizations: Tripartite-inactive alignment

In mixed domains like deverbal nominalizations, we expect the two alignments, i.e., syntactic ergativity from the clausal domain and split-genitive from the nominal domain, to interact in the way predicted by their internal structure (Alexiadou 2020; Iordăchioaia 2020a; Alexiadou et al. 2007 and references therein). In particular, the availability of verbal and nominal cases reflects the presence of verbal and nominal layers. For example, the availability of accusative case in English *-ing-*nominals correlates with the presence of an (accusative-assigning) transitive Voice head (26)a, whereas the unavailability correlates with the absence thereof (26)b (Alexiadou 2013; Harley 2009).

(26)
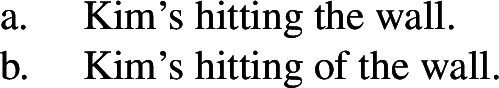

While distinct types of nominalization exist in Samoan, e.g., nominalizations derived by nominalizers like *-ga* or -C*aga*, our focus lies on bare nominalizations, i.e., nominalizations in which verbal predicates combine with an article in the absence of overt morphology (see Mosel and Hovdhaugen 1992; Mosel 1992, also Ball 2009 on Tongan, Cablitz 2000 on Marquesean, Massam 2000 on Niuean, Hooper 1996 on Tokelauan, cf. Moyse-Faurie 2016; van Lier and van Rijn 2013; Clark 1981; Chung 1973 for further discussion). Bare nominalizations thereby denote the eventuality of the nominalized verb. They have the same distribution as regular DPs but are often used as arguments of psych (27)a, perception (27)b, and communication verbs, or as headlines or (book/story) titles (27)c.

(27)
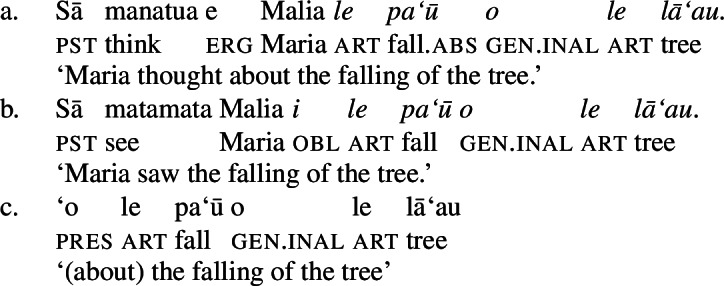

In the absence of absolutive case, bare nominalizations exhibit a tripartite-inactive alignment, in which all three types of subjects are marked by distinct case markers. Firstly, subjects of unaccusative verbs, including dynamic verbs like *pa‘ū* ‘fall’ and *tanu‘u* ‘arrive’ as well as stative verbs like *laulelei* ‘be beautiful’ are marked by inalienable genitive case.

(28)
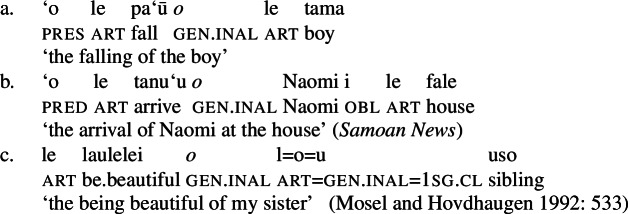

Secondly, subjects of unergative verbs like *pese* ‘sing’, *ata* ‘laugh’, and *galue* ‘work’ are marked by alienable genitive case. As a result, the choice of inalienable and alienable genitive case reflects the distinction between two types of intransitive subjects and can be used as a diagnostic for split-intransitivity (Collins 2014, cf. Burzio 1986; Perlmutter 1978; see Sect. 3.3 for further discussion).^14^

(29)
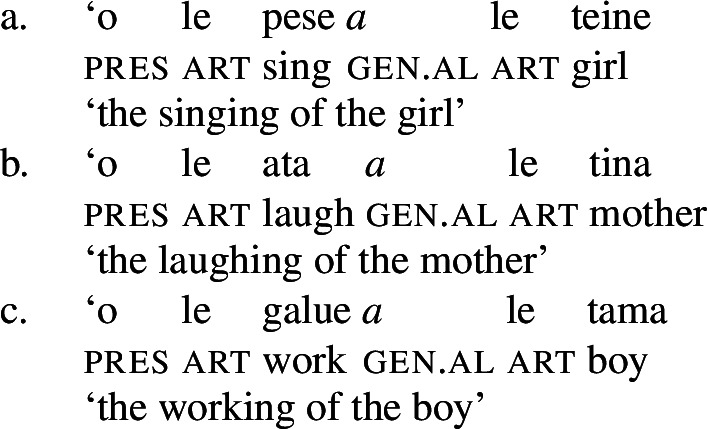

Finally, as expected from unaccusative nominalizations, objects of transitive verbs receive inalienable genitive case.^15^ Subjects of transitive verbs however maintain their ergative marking from the clausal domain and cannot appear with either alienable like unergative subjects or inalienable genitive case.

(30)
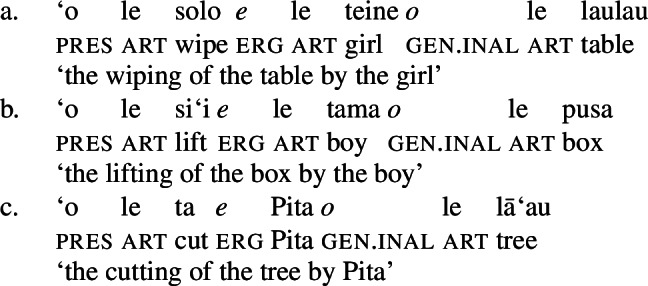

As mentioned by Muāgututi’a (2018), speakers often tend to insert the resumptive pro-form *ina* into bare nominalizations of transitive verbs; though its presence is not obligatory as such nominalizations without *ina* are frequently found in the literature (see Mosel and Hovdhaugen 1992) and judged grammatical by our consultants.^16^

(31)
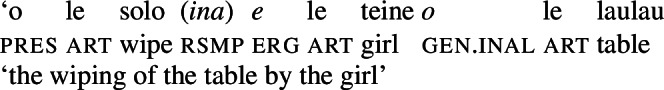

It is important to note that optional *ina* insertion is also observed outside of nominalizations in verbal clauses. Here, its distribution is sensitive to information structure properties, indicating the salience of the agent in the given discourse, if present (Mosel 1985, also Cook 1978).

(32)
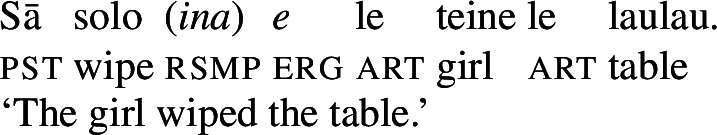

As in-situ *ina*-doubling is generally optional and not restricted to nominalizations, we take *ina-*doubling as an independent phenomenon without direct impact on the analysis of argument structure in nominalizations, especially in comparison to respective verbal clauses. While a detailed analysis of the morphosyntactic and pragmatic properties of *ina* doubling, especially in nominalizations, is far beyond the scope of this paper, we tentatively adopt a big-PP approach, in which *ina* and the ergative subject cooccur within a single PP, for explanatory purposes (Grewendorf 2008, cf. Cardinaletti 2019; Uriagereka 1995 for big XP approaches).^17^

(33)

To summarize, the combination of the ergative/absolutive alignment in the clausal domain and the split-genitive alignment sensitive to alienability in the nominal domain results in a tripartite-inactive alignment, in which all three types of subjects are marked by distinctly: unaccusative subjects and objects, are marked by inalienable genitive case, unergative subjects are marked by alienable genitive case, and transitive subjects maintain their ergative marking from the clausal domain (Table 1).

**Table 1:** Case marking in verbal clauses and nominalizations

	clausal domain	nominalization
S_unacc_	H_abs_	*o*_inal_
S_unerg_	H_abs_	*a*_al_
A	*e*_erg_	*e*_erg_
O	H_abs_	*o*_inal_

### Voice-under-*n*

To investigate the source of the tripartite-inactive alignment, we first establish the internal structure of Samoan bare nominalizations. Crosslinguistically, it has been argued that complex event nominalizations as mixed projections vary regarding the presence of nominal and verbal structure on the one hand, and the nominalizer, namely *n* or D, on the other hand (Alexiadou 2020; Iordăchioaia 2020a; Alexiadou et al. 2010). Crucially, the type of nominalizer determines the presence of nominal structure, as only *n*-based complex event nominalizations exhibit all properties of regular nouns (34), i.e., possessives, number, etc., whereas D-based complex event nominalizations lack lower nominal projections (35). Moreover, only D-based nominalizations can embed larger verbal structures, such as TPs or CPs.

(34)
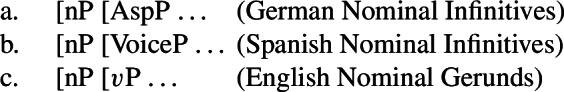

(35)
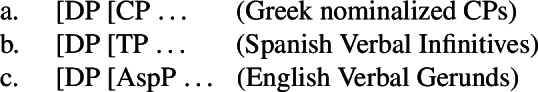

In the following, we demonstrate that Samoan bare nominalizations are *n*-based nominalizations that maximally embed AspP complements. As such, they are expected to obey the unaccusativity restriction on nominalizations, according to which DP arguments are banned from Spec, VoiceP under *n* (Alexiadou 2017, 2001; Imanishi 2020, 2014; Bruening 2013; Salanova 2007).

#### Verbal properties

Initial evidence for the presence of a *v*P in bare nominalizations comes from the availability of verbalizing morphology, such as the causative prefix *ta-* (36)a and the anticausative prefix *ma-* (36)b that form anticausative and causative variants from a-categorial roots (cf. Hopperdietzel 2020; Mosel and Hovdhaugen 1992: 184ff, 188f).

(36)
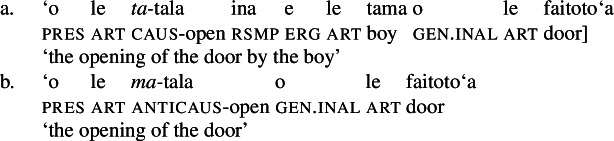

Likewise, the verbalizing prefix *liu-* which derives inchoative verbs from nouns, such as *liu-suavai* ‘become liquid, melt’ from *suavai* ‘(a) liquid’, can show up in bare nominalizations (Hopperdietzel 2020).^18^

(37)
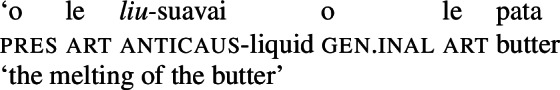

Further evidence comes from verbal agreement, marked by partial reduplication (38)a, designated plural morphology (38)b, or suppletion (38)c (Zuraw et al. 2014; Mosel and Hovdhaugen 1992: 220ff, 442ff). While the locus of plural agreement has been identified as *v* (Thornton 2019, cf. Bobaljik and Harley 2017; Toosarvandani 2016; Haji-Abdolhosseini et al. 2002), the verb agrees with the absolutive argument in verbal clauses but with the inalienable genitive argument in nominalizations (see also Sect. 2.4.2).^19^

(38)
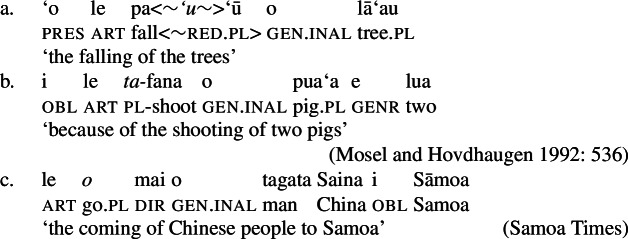

Moreover, Samoan verbs optionally indicate event number independently of argument number by pluractional morphology such as full reduplication (Thornton 2019; Mosel and Hovdhaugen 1992: 224ff, cf. Haugen 2011; Haji-Abdolhosseini et al. 2002), which is also grammatical in bare nominalizations.

(39)
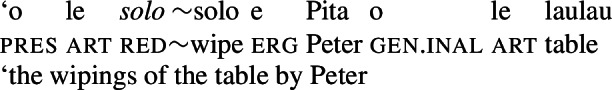

In addition to prefixed verbs, more complex directional *v*Ps can be nominalized where a motion verb combines with a directional particle like *ifo* ‘down’ which introduces a prepositional goal argument (see also example (38)c above; Mosel and Hovdhaugen 1992: 558).

(40)
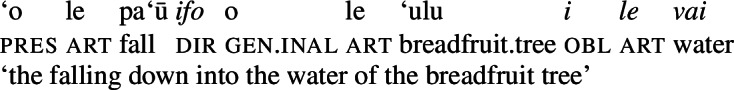

Finally, *v*P-internal modifiers like oblique causers are grammatical in nominalizations.^20^ The last two diagnostics indicate that bare nominalizations embed a phrasal *v*P (Hopperdietzel 2021, cf. Alexiadou et al. 2015).

(41)
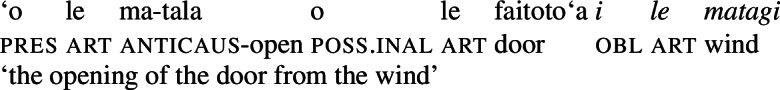

Evidence for the presence of a VoiceP in bare nominalizations comes from Voice-related morphology that cooccurs with ergative-marking on the subject (Hopperdietzel 2021, 2020; Tollan 2018): Firstly, the ergativizing suffix -C*ia* which derives ergative clauses from verbs (42)a that cannot take an ergative subject otherwise (42)b, adding a higher degree of agency (cf. Cook 1996; Mosel and Hovdhaugen 1992: 198ff).

(42)
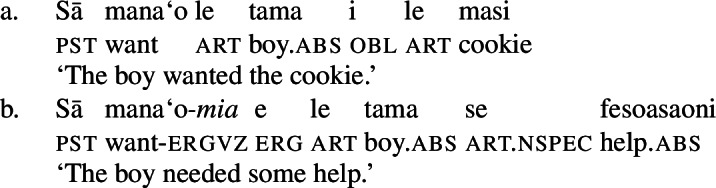

Secondly, the causative prefix *fa‘a-* obligatorily introduces (ergative) agentive causers (43)a but is ungrammatical with (oblique) nonagentive causers (43)b (Hopperdietzel 2021; Koopman 2012, cf. Mosel and Hovdhaugen 1992: 175f).

(43)
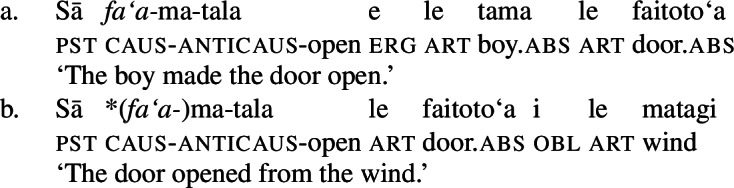

That both types of Voice-related morphology are grammatical in bare nominalizations indicates that bare nominalizations are derived from a verbal core which minimally includes a VoiceP.

(44)
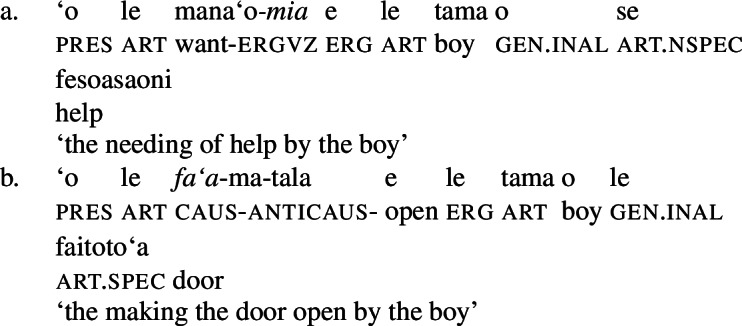

Additional support for this analysis comes from the availability of instrumental modification in bare nominalizations, as illustrated by the modifier *i lona lima tusi* ‘with its forefinger’ below.

(45)
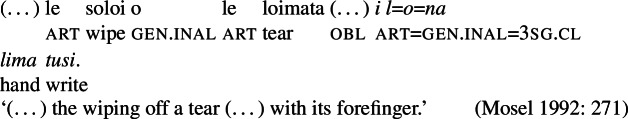

Further, the presence of post- and preverbal aspectual modifiers suggests that the nominalized verbs can also come with aspectual layers (Mosel and Hovdhaugen 1992: 381ff, 557ff). This holds for postverbal aspectual modifiers like *so‘o* ‘frequently’ (46), as well as for preverbal aspectual modifiers like *toe* ‘again’ (47). In nominalizations, the latter occur in between the article and the verb.

(46)
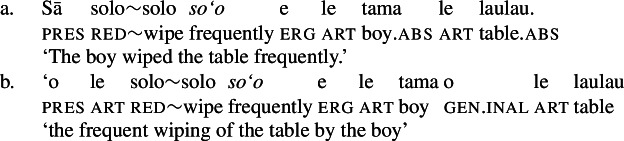

(47)
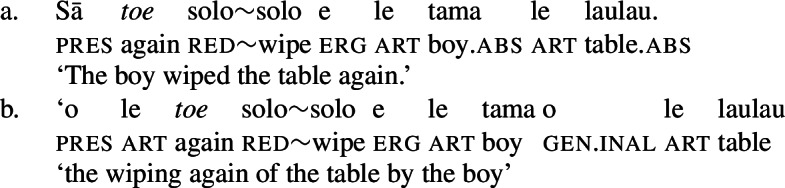

While the presence of (low) aspectual adverbs signals the availability of aspectual projections in bare nominalizations (cf. Cinque 1999; Alexiadou 1997), bare nominalizations do not embed constituents of the size of a TP. Unlike certain types of nominalizations in Turkish and Japanese (Kornfilt and Whitman 2012), tense morphology such as the past tense marker *sā* is ungrammatical in Samoan (Collins 2014).

(48)
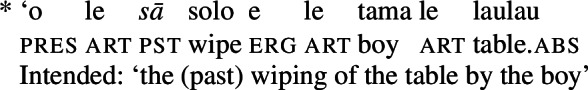

As absolutive case is associated with a TP-layer in Samoan (Tollan 2018; Koopman 2012), its absence supports a TP-less analysis (*pace* Collins 2014, cf. de Miguel 1996 on nominative case in Spanish verbal infinitives).

(49)
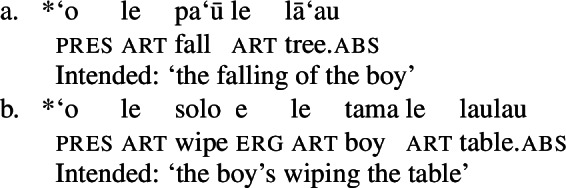

As expected by the absence of a TP-layer, also higher sentential adverbs that occur to the left of tense/aspect particles (50) as well as complementizers are ungrammatical in bare nominalizations (51) (Collins 2014).

(50)
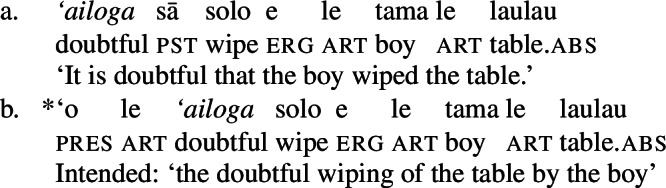

(51)
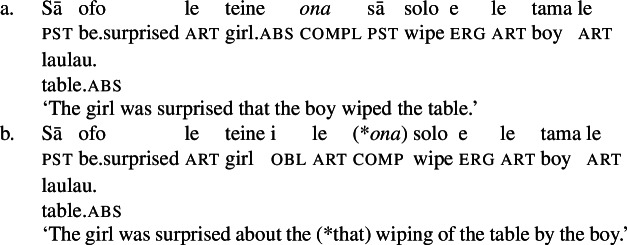

Summarizing our investigation (Table 2), bare nominalizations embed a reduced verbal structure of maximally the size of an Asp(ectual)P, with higher projections such as TP and CP generally absent. This indicates that Samoan bare nominalizations are complex event nominals that come with a verbal argument structure.

**Table 2:** Verbal layers in Samoan bare nominalizations

Layer	Verbal clauses	Nominalizations	Diagnostics
***v***P	Yes	Yes	– verbalizing morphology
			– number agreement
			– pluractionality
			– directionals
			– (oblique) causers
VoiceP	Yes	Yes	– ergativizing morphology (-C*ia*)
			– causative morphology (*fa‘a-*)
			– instrumental modification
			– ergative marking
AspP	Yes	Yes	– postverbal aspectual modifiers
			– preverbal aspectual modifiers
TP	Yes	No	– T/A marker
			– absolutive case
CP	Yes	No	– sentential adverbs
			– complementizer

#### Nominal properties

Based on the morphosyntactic type of the nominalizer, two types of nominalizations have been proposed crosslinguistically (Alexiadou 2020; Iordăchioaia 2020a; Alexiadou et al. 2010). On the one hand, *n*-based nominalizations exhibit all nominal layers and thus the properties of regular nouns, including possessives, diminutives, number, and different types of determiners (52)a. On the other hand, D-based nominalizations are somewhat defective as they only include a D-layer with projections for number, diminutives, and possessives missing (52)b.

(52)
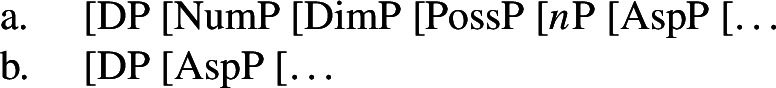

We now demonstrate that Samoan bare nominalizations pattern with *n*-based nominalizations, even though the nominalizer itself is not overtly realized. Initial evidence comes from alienable genitive case on subjects of unergative nominalizations, which is associated with the presence of a PossP in the nominal domain (see Sect. 2.2.2; Tyler 2021; Alexiadou 2001).^21^

(53)
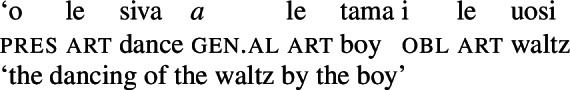

In Samoan, nominal properties are usually realized on the prenominal determiner (Mosel and Hovdhaugen 1992: 149): Firstly, the article *si* is used for diminutives which are “implicitly specific and connotes sympathy and belittlement” (Mosel and Hovdhaugen 1992: 264). Notably, the diminutive article can combine with both regular nouns (54)a and bare nominalizations (54)b (Mosel and Hovdhaugen 1992: 538).

(54)
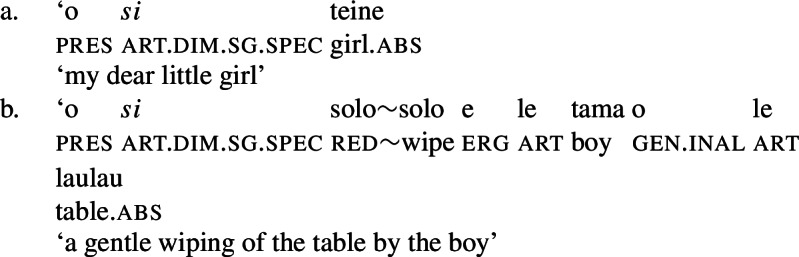

Secondly, number marking of specific plural noun phrases is indicated by the absence of an overt article. Again, both regular nouns (55)a and nominalizations (55)b can appear in their bare forms; though plural marking on the latter is rather rare with speakers preferring verbal plural marking instead (Mosel and Hovdhaugen 1992: 539).^22^

(55)

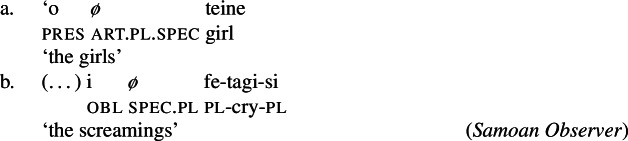

Finally, nominalizations cannot only be combined with specific articles but also with nonspecific articles like the singular *se*, primarily in negative and irrealis contexts (Mosel and Hovdhaugen 1992: 537f).

(56)
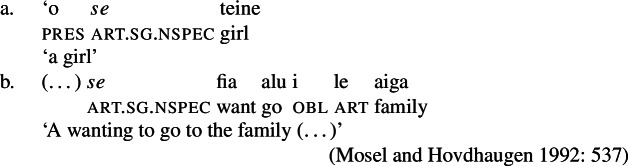

The observation that different types of determiners can occur in Samoan bare nominalizations therefore provides support for *n*-based analysis, since D-based nominalizations have been argued to be restricted to default determiners, due to their defective nature (i.e., the lack of D-*n* agreement; Iordăchioaia 2014).

Depending on their syntactic position, bare nominalizations can take absolutive case (27)b or combine with oblique (27)b, and more rarely ergative (57), prepositions, further supporting the assumption of the presence of a DP layer in bare nominalizations.

(57)
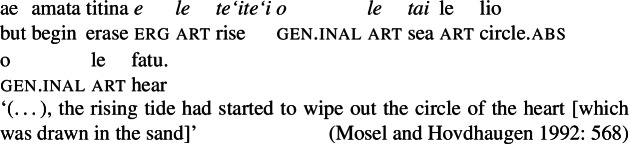

To summarize, bare nominalizations show the same morphosyntactic properties as regular DPs and can thus be classified as *n*-based nominalizations that entail all nominal layers, including PossP, DimP, NumP, and DP (Table 3).

**Table 3:** Nominal layers in Samoan bare nominalizations

Layer	Regular nouns	Nominalizations	Diagnostics
*n*P	Yes	Yes	– variable determiners (via D-*n* agreement)
PossP	Yes	Yes	– alienable genitive case on unergative subjects
DimP	Yes	Yes	– diminutive article *si*
NumP	Yes	Yes	– plural article ø
DP	Yes	Yes	– determiners
			– case marking
			– prepositions

#### The unaccusative requirement on n

Based on their morphosyntactic properties, we can classify Samoan bare nominalizations as *n*-based complex event nominals that embed verbal constituents maximally of the size of AspP.

(58)

In the following, we first focus on nominalizations embedding a VoiceP complement, before returning to nominalizations embedding an AspP in Sect. 3.3. Crosslinguistically, Voice-under-*n* in many languages have been argued to be subject to an unaccusative requirement (59) (Alexiadou 2017, 2001; Imanishi 2014; Bruening 2013; Salanova 2007, but see Šereikaitė 2023; Imanishi 2020; Smirnova and Jackendoff 2017 for a more nuanced view), i.e., *n* selects for VoiceP complements that lack a specifier. Specifically, Bruening (2013) argued in detail that *n* in nominalization is similar to a passive head: next to changing lexical category it also demotes the external argument, which may then be introduced as an adjunct in the verbal domain, i.e., a *by*-phrase,^23^ or as a possessor argument in Spec, PossP in the nominal domain.^24^

(59)
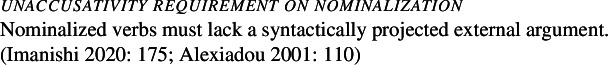

This restriction has consequences for both transitive and unergative subjects which are merged *v*P-externally in Spec, VoiceP, as illustrated by Greek: Transitive subjects, on the one hand, can only be realized by an optional *by*-phrase. Consequently, even languages that display an accusative alignment in the clausal domain show an ergative alignment in the nominal domain (see Alexiadou 2017 for a detailed discussion and crosslinguistic data).

(60)

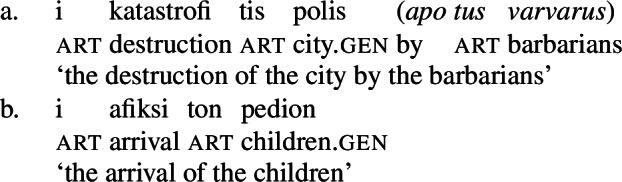

Unergative subjects, on the other hand, are merged as possessors in the nominal domain, as they violate the unaccusative requirement in the absence of a prepositional (passive) alternative. They therefore differ from transitive and unaccusative subjects in not being thematically licensed within the verbal domain, as indicated by the infelicity of agent-oriented modifiers like *siniditi* ‘conscious’ in (61).^25^

(61)

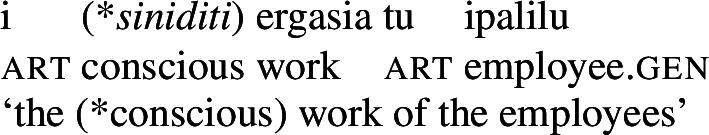

As a result, the unaccusative requirement on *n* determines how external arguments can be introduced in nominalizations, namely either as oblique *by*-phrases in the verbal domain in the context of nominalizations of transitive verbs or as a possessor in the nominal domain in the context of unergative verbs.^26^

### Three types of subjects

In the following, we demonstrate how the tripartite-inactive alignment in Samoan nominalizations follows naturally from the interaction of the unaccusative requirement on *n*-based nominalizations with the split-genitive marking based on (in)alienability and prepositional ergativity.

#### Unaccusatives

As *v*P-internal arguments, unaccusative subjects obey the unaccusative requirement on nominalizations and are merged in their canonical position as complements of *v* below an unaccusative expletive Voice head in both the clausal domain and bare nominalizations. In the absence of T in nominalizations, unaccusative subjects do not receive absolutive case but inalienable genitive case, the unmarked case in the nominal domain (cf. Alexiadou 2017; Baker 2015).

(62)
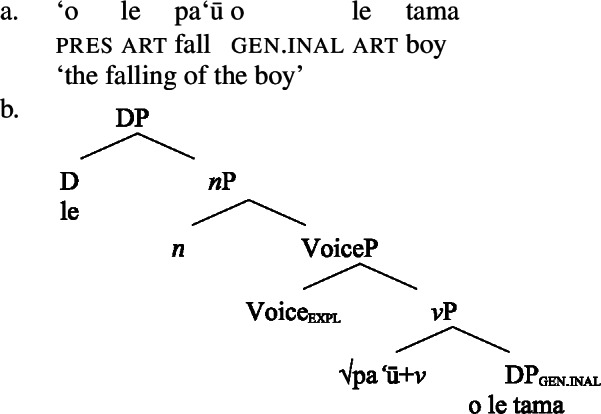

#### Unergatives

As *v*P-external arguments, unergative subjects violate the unaccusative requirement on nominalizations, as they are merged in Spec, VoiceP in the clausal domain (Alexiadou et al. 2015; Kratzer 1996). In nominalizations, unergative subjects are therefore merged as possessors in Spec, PossP in nominalizations to satisfy the unaccusative requirement. Consequently, unergative subjects receive inherent alienable genitive case from Poss instead of unmarked inalienable case like unaccusative subjects. Unlike in languages like Greek or English, the two types of intransitive subjects are therefore distinguished due to the split-genitive alignment in the nominal domain.

(63)
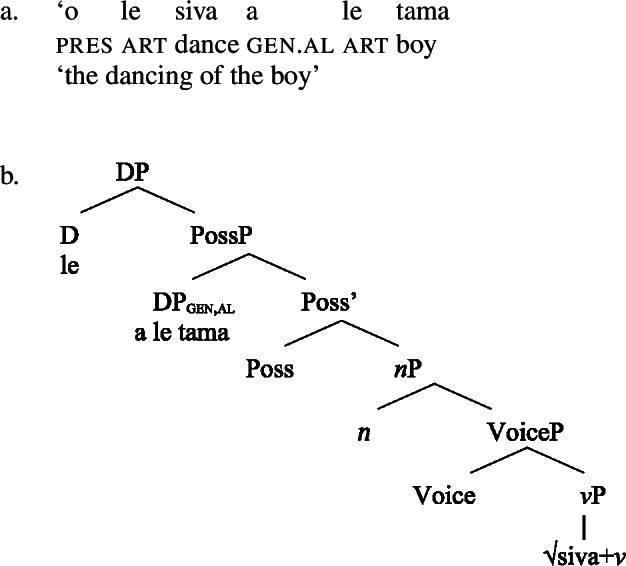

Crucial evidence for the special status of unergative subjects in nominalizations comes from the observation that unlike unaccusative subjects, number agreement (via partial reduplication; see Sect. 2.3.1) on nominalized unergative verbs is strongly disfavored (64)b; although it is grammatical in verbal clauses (64)a. As number agreement has been argued to be subject to locality constraints (Thornton 2019; Bobaljik and Harley 2017), this contrast suggests that unergative subjects in nominalizations appear in a nonlocal position to *v*, which we identify as Spec, PossP.

(64)
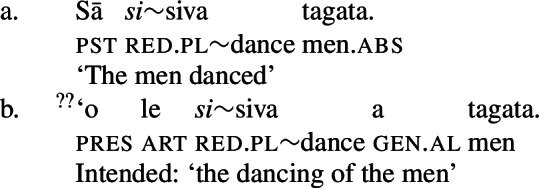

However, nominalized unergative verbs still exhibit a verbal core, as they allow full reduplication to mark pluractionality (65) and take hyponymous (66)a and cognate objects (66)b, which in Samoan are introduced as oblique arguments (cf. Tollan and Massam 2022; Tollan 2018 for a discussion).^27^

(65)

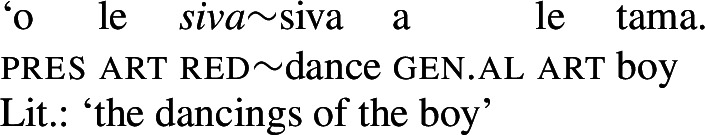

(66)
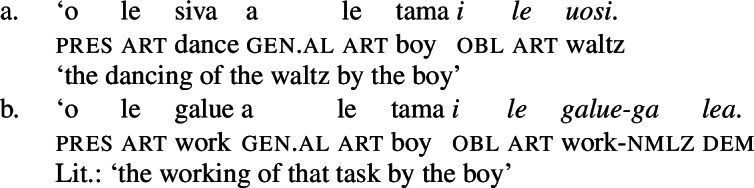

Although the unergative subject is introduced in the nominal domain, nominalized unergative verbs still contain an (unaccusative) agentive Voice head, as they cooccur with Voice-related morphology, such as the causative prefix *fa‘a-* (67)a and the ergativizing suffix *-mia* (67)b, e.g., in the context of object omission or pseudo noun incorporation (cf. Mosel 1992).^28^

(67)
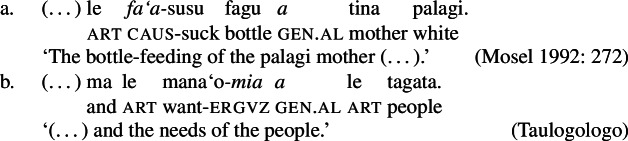

In nominalizations, unergative subjects therefore differ from unaccusative subjects in that they are merged as possessors outside of the verbal domain, in Spec, PossP, where they receive inherent alienable genitive case, resulting in morphological distinct marking.

#### Transitives

As discussed in Sect. 2.2.1, transitive subjects in a syntactically ergative language like Samoan are merged as PPs, resembling passive *by*-phrases in accusative languages like English and Greek. As such, transitive subjects in Samoan satisfy the unaccusative requirement on nominalization since Spec, VoiceP is not occupied. Therefore, ergative marking survives nominalization in Samoan. In the absence of absolutive case, objects as internal arguments receive unmarked inalienable genitive case, parallel to unaccusative subjects, giving rise to the observed tripartite-inactive case alignment.^29^

(68)
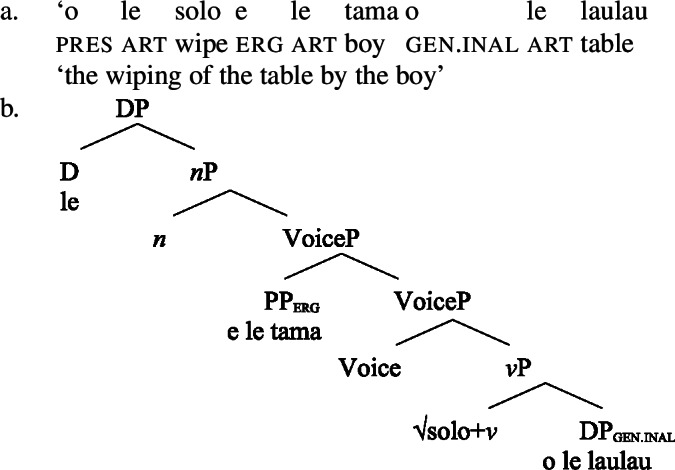

However, if transitive subjects bear absolutive case, e.g., due to pseudoincorporation (69) or antipassivization of the object (70) (Collins 2017; Shibatani 2006, cf. Mosel and Hovdhaugen 1992: 108ff, 738ff), transitive and unergative subjects behave alike, i.e., being introduced as possessors marked by alienable genitive *a* case (Mosel 1992).

(69)

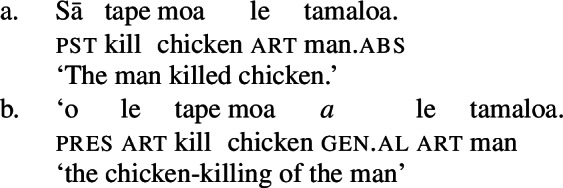

(70)
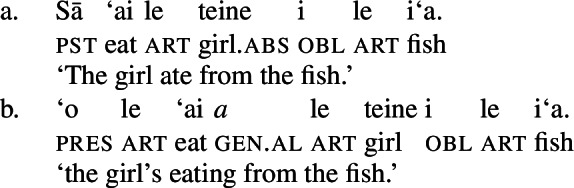

The examples above therefore suggest that the grammaticality of ergative marking in bare nominalizations follows from its prepositional nature, as external DP-arguments become ungrammatical in such contexts, providing novel crosslinguistic evidence for the unaccusative requirement on nominalizations.

To summarize, we derive the tripartite-inactive alignment from a language-specific combination of independent phenomena: (i) split-intransitivity, (ii) split (in)alienability, (iii) prepositional ergativity, and (iv) the unaccusative requirement on nominalizations. Unergative DP subjects are thus merged in the nominal domain, receiving alienable genitive case, whereas transitive subjects can maintain their prepositional ergative marking. In the absence of absolutive case, internal arguments, i.e., unaccusative subjects and objects, get unmarked inalienable genitive case.

## Marked unergatives

Further evidence for the special status of unergative subjects in bare nominalizations comes from subject clitics, which in the clausal domain show a neutralized alignment. In nominalizations, both unaccusative and transitive subject clitics receive inalienable genitive case, whereas unergative subject clitics receive alienable genitive case (Mosel and Hovdhaugen 1992; Mosel 1992). Motivating a nonuniform analysis of Samoan clitic pronouns (cf. Bleam 2000; Uriagereka 1995; Sportiche 1996, see Anagnostopoulou 2017 for an overview), we take intransitive subject clitics to be derived by movement out of a big-DP, where they are assigned their respective case. In contrast, transitive subject clitics are merged directly to a higher syntactic head, being resumed by prepositional *ina* in their original theta position in Spec, VoiceP, parallel to left dislocation of regular transitive subjects. As a result, transitive subject clitics are exceptionally marked by the unmarked case of the respective domain, i.e., absolutive in the clausal and inalienable genitive in the nominal domain (cf. Baker 2015; Cardinaletti and Starke 1999). We therefore derive the distinct marking of unergative subjects again from the unaccusative requirement on nominalizations, as only unergative subject clitics require both case and thematic licensing in the nominal domain.

Towards the end of the section, we also address apparent variation in genitive marking of intransitive subjects, i.e., alienable genitive case on unaccusative subjects and inalienable genitive on unergative subjects. We demonstrate that this alternation has syntactic and semantic effects, which further supports our analysis of Samoan bare nominalizations and provides novel evidence for a variable intransitivity (cf. Neu 2023; Sorace 2000).

### Subject clitics

We begin our investigation of marked unergatives with an investigation of preverbal subject clitics, which exhibit a neutralized alignment with all three types of subjects being marked by absolutive case. Based on the distribution of the resumptive pronoun *ina* (Mosel 1985; Cook 1978), we argue that transitive subject clitics do not result from movement out of a big-DP but are directly merged in the inflectional domain (cf. Bleam 2000).

#### Subject clitics vs. independent pronouns

In verbal clauses, clitic pronouns in Samoan appear in between T/A markers and the verbal predicate, typically in the second position of the clause (with the exception of the generic T/A-marker which precedes the clitic), realizing the person and number feature of (in)transitive subjects (Cook 1994; Mosel and Hovdhaugen 1992: 455ff, cf. Moyse-Faurie 1997 for a cross-Polynesian overview).^30^ While unaccusative and unergative subject clitics maintain their absolutive case in preverbal position (71)a/b, transitive subject clitics are not marked by ergative but by absolutive case instead (71)c. Therefore, subject clitics exhibit a neutralized alignment, including double absolutive case in the context of transitive verbs (see Yu 2021 for a discussion of tonal absolutive on preverbal subject clitics). Similar to left dislocation of transitive subjects, ergative drop on preverbal transitive subject clitics shows a strong tendency to cooccur with the prepositional resumptive pronoun *ina* (also Cook 1996; Chung 1978).^31^

(71)
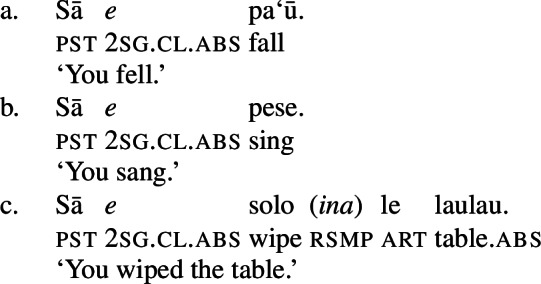

Phonologically, subject clitics are reduced independent personal pronouns, which appear in postverbal position and pattern like regular DPs showing an ergative alignment; though third-person singular pronouns tend to be marked by the presentative marker *`o*, when they are appear as absolutive arguments, as illustrated by the object in (72)c (Mosel and Hovdhaugen 1992: 121f).

(72)
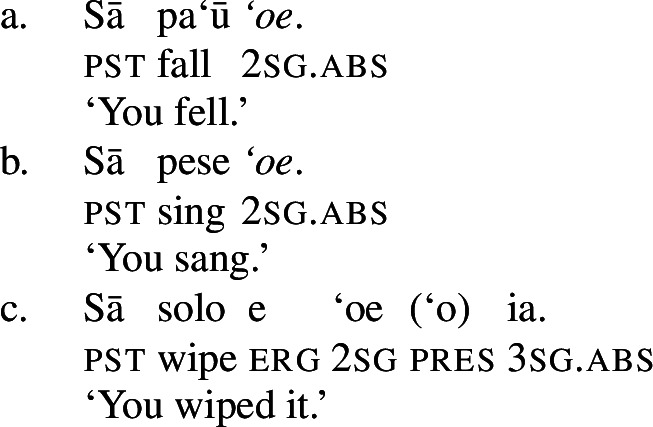

Table 4 provides a (simplified) overview of the morphophonological relations between different pro-forms, indicating that the plural forms differ from the singular and dual forms in having the same phonological form for both clitic and independent pronouns (Mosel and Hovdhaugen 1992: 121f).^32^ Our examples will primarily come from singular contexts, leaving potential variation between singular and plural clitics to future research.

**Table 4:** Independent and clitic pronouns in Samoan (adapted from Mosel and Hovdhaugen 1992: 121f)

	sg		dual		pl	
	Independent	Clitic	Independent	Clitic	Independent	Clitic
1.incl	*a‘u*	*ou / =u*	*ta‘ua*	*tā*	*tatou*	*tātou*
1.excl	*—*	*—*	*ma‘ua*	*mā*	*matou*	*mātou*
2	*‘oe*	*e*	*‘oulua*	*lua*	*‘outuo*	*‘outuo*
3	*ia*	*ia, na*	*la‘ua*	*lā*	*latuo*	*lātuo*

Subject clitics can be identified as true clitics (in the sense of Cardinaletti and Starke 1999), as they can be doubled by independent pronouns in postverbal position, which gives to an emphatic reading related to (contrastive) focus (Mosel and Hovdhaugen 1992: 457, cf. Moyse-Faurie 1997).^33^

(73)
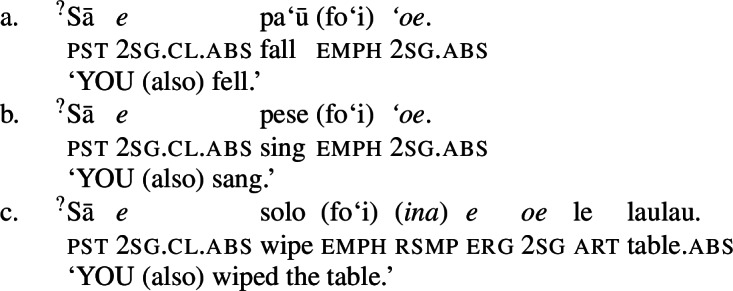

In transitive contexts (73)c, this can result in clitic tripling with the subject expressed by the preverbal absolutive subject clitic as well as the postverbal prepositional pro-form *ina* and the ergative independent pronoun.

#### A nonuniform analysis of subject clitics

We tentatively adopt a nonuniform analysis of subject clitics in Samoan, according to which transitive subject clitics are similar to agreement markers being merged directly to T, whereas intransitive subject clitics are derived by movement out of a big-DP (see Bleam 2000 on the movement vs. base-generation distinction in Spanish accusative and dative clitics, cf. Anagnostopoulou 2017; Cuervo 2003). This analysis is motivated by the parallelism of dislocated transitive subjects: In both left dislocation (74)a and cliticization (74)b, the transitive subject appears without ergative marking but the postverbal prepositional resumptive pronoun shows up in the postverbal subject position.^34^

(74)
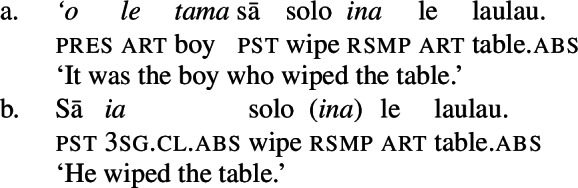

In Sect. 2.2.1, we have developed an analysis of dislocated transitive subjects as being base-generated in clause-initial position, being resumed by *ina* in the original subject position, due to their prepositional nature prohibiting their movement. Adopting a base-generation account (Baker and Kramer 2018, cf. Sportiche 1996*inter alia*, also Polinsky 2016 on Tongan), we propose a related account for transitive subject clitics. In particular, we assume that transitive subject clitics are merged directly to T, where they, as D-elements, receive unmarked absolutive case and undergo T-to-C movement. In this position, they are resumed overtly by the prepositional *ina*, and sometimes covertly by *pro* (see FN31), in the thematic subject position in Spec, VoiceP (see Polinsky 2016 on *pro* resuming preverbal clitics in Tongan).^35^

(75)
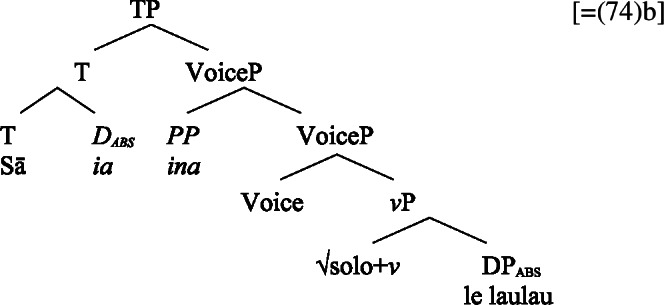

For intransitive subject clitics, we again notice the parallelism to left dislocation, suggesting that preverbal clitic intransitive subjects clitics do not require resumption in such contexts.

(76)
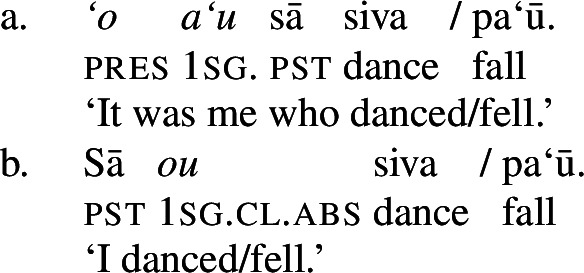

We therefore adopt a movement analysis of intransitive subject clitics (Cardinaletti 2019; Grewendorf 2008; Uriagereka 1995 inter alia, cf. Anagnostopoulou 2017 for an overview, also Collins 2017 on Samoan). Accordingly, intransitive subject clitics are base-generated together with an independent pronoun within a big-DP, where they both are assigned absolutive case before moving to T, as illustrated for unergative (77)a and unaccusative subject clitics (77)b below.

(77)
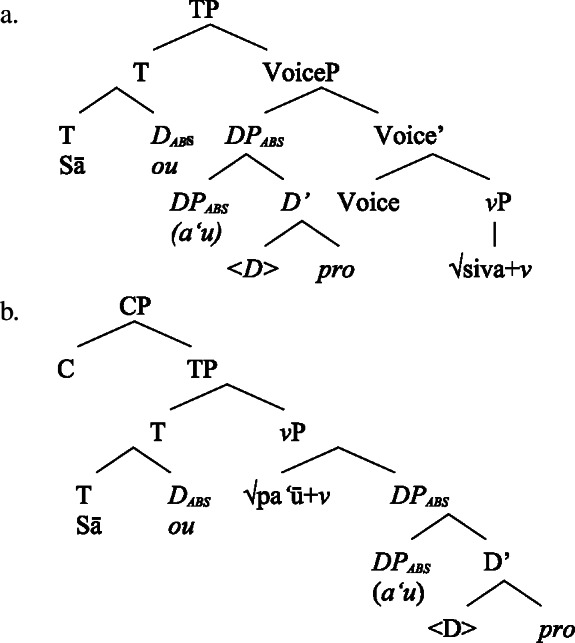

To summarize, we proposed a nonuniform analysis of Samoan subject clitics which follows from the prepositional nature of ergative subjects: Transitive subject clitics are base-generated in T and resumed by the prepositional pronoun *ina*, whereas intransitive subject clitics are born within a big-DP in the original argument position before moving to T.

### Possessive clitics

The distribution of clitics is not limited to the verbal domain as they also occur as prenominal possessive clitics in the nominal domain, where they are transparently marked by either alienable or inalienable genitive case (Mosel 1992; Mosel and Hovdhaugen 1992: 549ff). Subject and possessive clitics exhibit the same morphophonological form, except for the second-person singular, which has the suppletive form =*u*, and the third-person singular, which is restricted to *na* in prenominal position (Table 5). If a clitic starts with a vowel, the initial vowel is fused with the genitive case marker *a* or *o*. Given those minor morphophonological differences, we take subject and possessive clitics to instantiate the same underlying phenomenon which primarily differ in their case marking.

**Table 5:** Subject and possessive clitics in Samoan (adapted from Mosel and Hovdhaugen 1992: 124)

	sg		dual		pl	
	Subject	Possessive	Subject	Possessive	Subject	Possessive
1.incl	*o‘u / =u*	*=‘u*	*tā*	*=tā*	*tātou*	*=tātou*
1.excl	*—*	*—*	*mā*	*=mā*	*mātou*	*=mātou*
2	*e*	=*u*	*lua*	*=lua*	*‘outuo*	*=utou*
3	*ia, na*	*=na*	*lā*	*=lā*	*lātuo*	*=lātuo*

In the following, we show that possessive clitics exhibit the same (in)alienable split as possessors realized by regular DPs and independent pronouns. In contrast to the neutralized alignment of preverbal subject clitics in the clausal domain and tripartite-inactive alignment of regular DPs and independent pronouns in bare nominalizations, we observe a marked unergative alignment, in which only unergative clitics receive alienable genitive case. We then demonstrate how this unexpected pattern supports our analysis of bare nominalizations in Samoan.

#### (In)alienability split

Like regular DPs, independent possessive pronouns that appear in postnominal position are marked for either alienable or inalienable genitive case, depending on the possessive relation between the possessor and the possessum (Mosel and Hovdhaugen 1992: 280).

(78)
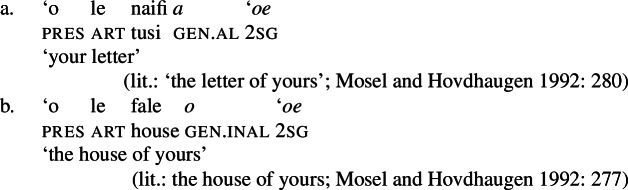

Although independent possessive pronouns are available, speakers usually prefer the use of prenominal possessive clitics, which exhibit the same (in)alienability split as other possessive noun phrases (Mosel and Hovdhaugen 1992: 278). Phonologically, possessive clitics clitisize with their genitive case marker and the article.^36^

(79)
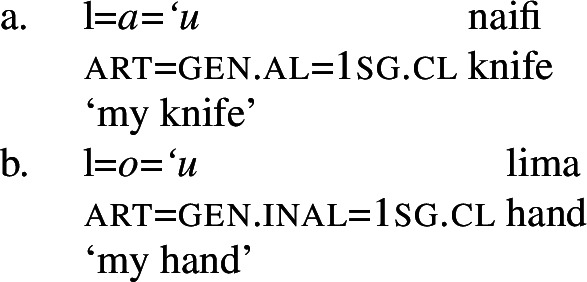

Building on our analysis of subject clitics, we propose that possessive clitics are base-generated in-situ within a (silent) big-DP where they are assigned the respective genitive case, before moving to a higher position. Inalienable possessive clitics are therefore born in the complement position of *n*, where they are assigned unmarked inalienable genitive case (compare Sect. 2.2.2; cf. Baker 2015; Myler 2016; Alexiadou 2001). Parallel to the verbal domain, possessive clitics as defective elements move to D (Alexiadou et al. 2007; Cardinaletti 1998).^37^

(80)
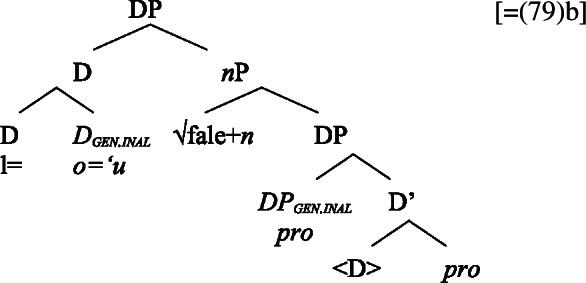

Alienable possessive clitics are introduced in Spec, PossP, where they are assigned inherent alienable genitive case by Poss before moving to D.

(81)
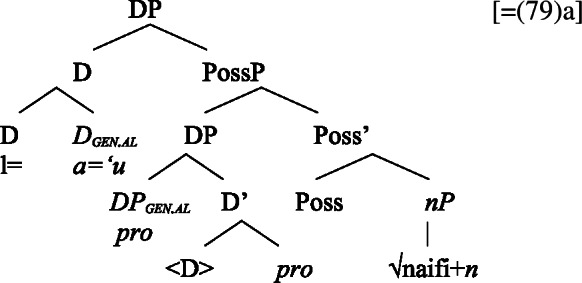

The (in)alienability split of possessive clitics therefore reflects the distinct syntactic positions in which possessive clitics enter the derivation: While clitics bearing inalienable genitive case are base-generated in the complement position of *n*, clitics bearing alienable genitive case are base-generated in Spec, PossP.

#### Possessive clitics in nominalizations

In bare nominalizations, postverbal independent pronouns exhibit the same tripartite-inactive alignment as regular DPs with unaccusative subjects (82)a and objects being marked by inalienable genitive, unergative subjects by alienable genitive case (82)b, and transitive subjects by the ergative (82)c.

(82)
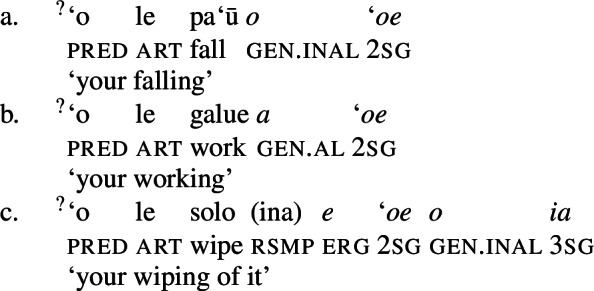

As expected, speakers generally prefer the use of possessive clitics in bare nominalizations, obligatorily referring to the subject of the nominalized predicate (cf. Mosel and Hovdhaugen 1992: 549ff, Mosel 1992). Instead of tripartite-inactive alignment of regular DPs and independent pronouns in bare nominalizations and the neutralized alignment outside of nominalizations, possessive clitics exhibit a crosslinguistically rare marked unergative alignment (again not mentioned by Comrie 2013): While transitive subject clitics (83)c receive inalienable genitive case like unaccusative subject clitics (83)a, unergative subject clitics are distinctly marked by alienable genitive case (83)b. Nominalized transitive verbs therefore exhibit a double inalienable genitive alignment (83)c.

(83)
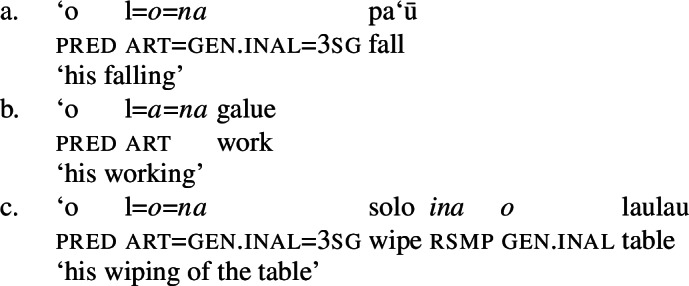

Building on our analysis of regular DP arguments, we take unaccusative subject clitics (83)a to be base-generated within a big-DP in *v*P-internal position, where they get unmarked inalienable genitive case before moving to D.

(84)
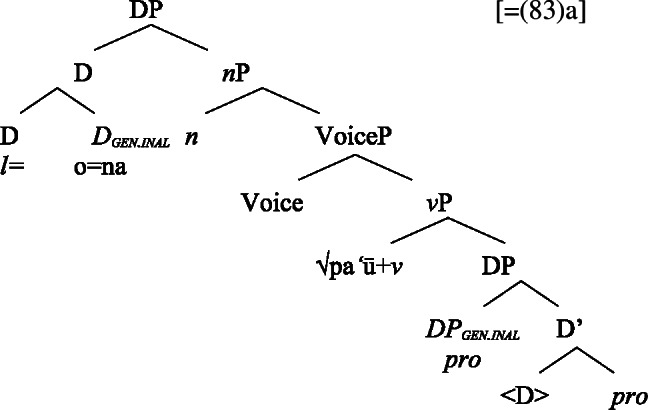

While unaccusative subject clitics obey the unaccusative requirement on nominalizations, unergative subject clitics (83)b must be introduced outside of the verbal domain, namely as possessors in Spec, PossP in the nominal domain. In this position, they receive inherent alienable genitive case as part of a big-DP and move to D.

(85)
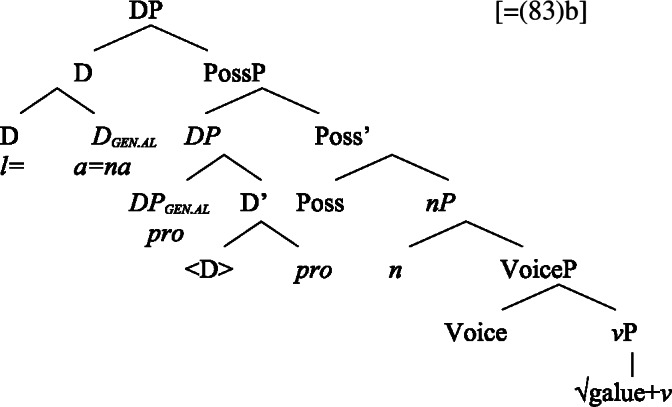

For transitive subject clitics (83)c, we note that parallel to the clausal domain, inalienable marking cooccurs with *ina* in postverbal subject position. Applying our nonuniform analysis of subject clitics to nominalizations, transitive subject clitics are merged outside of the verbal domain directly to D, where they receive unmarked inalienable genitive case, in local configuration with D (cf. Baker 2015). Being base-generated in an athematic position, prepositional *ina* resumes the transitive subject clitic in Spec, VoiceP, in line with the unaccusative requirement. Objects likewise receive unmarked inalienable genitive case, resulting in a double inalienable genitive alignment.^38^

(86)
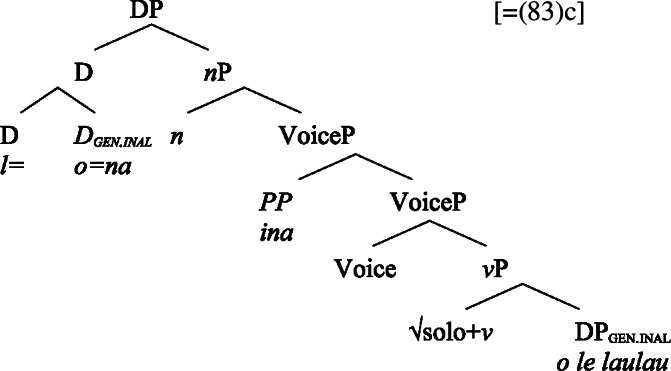

The distinct marking of unergative subjects in Samoan bare nominalizations therefore follows naturally from a combination of the unaccusative requirement on nominalizations, split-(in)alienability marking of possessors, and a nonuniform syntax of subject clitics in a prepositional ergative language like Samoan.

#### Agents vs. possessors

As unergative and transitive subjects are assigned distinct thematic roles, i.e., agent in Spec, VoiceP and possessor in Spec, PossP, we predict interpretative differences between the two types of subject, since the possessor relation has been argued to be generally underspecified, allowing for various pragmatically salient interpretations (cf. Harley 2009: 324, Alexiadou 2001; Barker 1995). This prediction is borne out by the data: Alienable genitive marking on unergative subjects gives rise to additional readings, including an ownership reading, either associated with a specific way in which the event is performed or a recording of the event (which for some speakers is the reading most readily available; cf. Hooper 2000, 1996 on nominalizations in closely related Tokelauan).

(87)
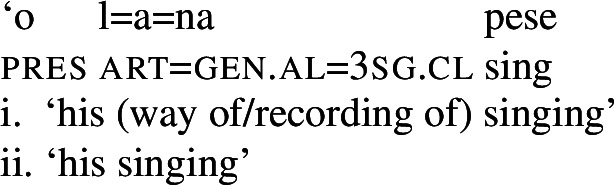

Inalienable transitive subjects crucially lack such readings and must instead be interpreted as event participants, i.e., agents, as a consequence of thematic linking via *ina* resumption (88)a. The same holds for unaccusative subjects which obligatorily receive a patient interpretation. To express an ownership reading with transitive nominalizations, the object must undergo pseudo noun incorporation (88)b, which then requires the subject to be merged in Spec, PossP to satisfy the unaccusative requirement, as illustrated by the contrast below.

(88)
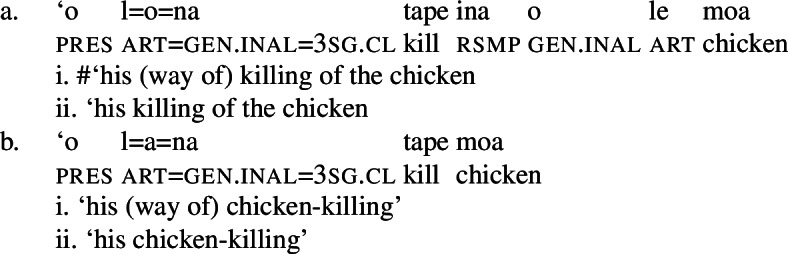

As transitive subject clitics receive their agent role already in the verbal domain via *ina* resumption, they are blocked from being merged in another theta position such as PossP, violating the theta criterion (Chomsky 1981). D as an athematic clitic-hosting position is therefore the only option for transitive subject clitics to be introduced, ruling out alienable genitive case on transitive subject clitics. The observed interpretative differences between inalienable genitive and alienable genitive possessors in nominalizations therefore provide independent support for our assumption that inalienable genitive case is the unmarked case in the nominal domain that is not associated with a specific theta role, whereas alienable genitive case is inherently assigned to alienable possessors.

### Variable intransitivity

Our discussion of the distribution of genitive cases in Samoan nominalizations so far suggests that genitive case assignment on intransitive subjects is categorial, with unergative subjects being marked by alienable and unaccusative subjects by inalienable genitive case. However, as already noted by Chung (1978) and Mosel (1992), genitive case can alternate on intransitive subjects. In the following, we demonstrate that these alternations are semantically meaningful and reflect distinct syntactic positions in which intransitive subjects can be merged (cf. Neu 2023; Tollan 2018; Massam 2009; Sorace 2000), which further supports our analysis.

#### Alienable genitive case on unaccusative subjects

Although unaccusative subjects are usually marked by inalienable genitive case, they can also receive alienable genitive case. Crucially, the case alternation has a semantic effect on the interpretation of the genitive argument: While inalienable-genitively marked arguments are obligatorily interpreted as event participants, i.e., patients (89)a, alienable-genitively marked arguments are interpreted as possessors (89)b.

(89)
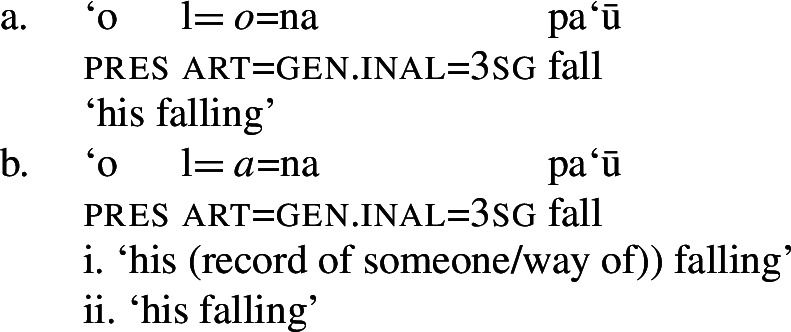

Parallel to unergative subjects (see Sect. 3.2.3), alienable-genitive marking therefore gives rise to a more flexible possessor relation, in which the genitive argument can be also interpreted as the owner of the event, e.g., a record of a falling event on social media, or as the creator of a specific manner/method of falling, e.g., in high diving. In addition to the verb-internal position, nominalizations of typically unaccusative verbs thus allow their internal argument to be merged in Spec, PossP in the nominal domain, parallel to unergative verbs.^39^

(90)
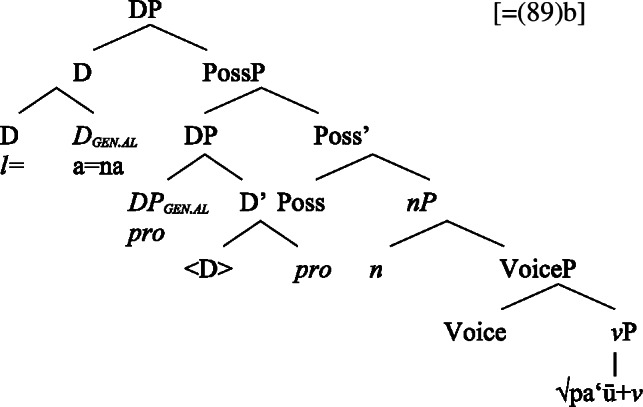

The variation of genitive marking in Samoan nominalizations thereby resembles variation in unaccusativity diagnostics, observed crosslinguistically, e.g., the choice of perfect auxiliaries in Italian (Sorace 2000). In general, alienable genitive case on the sole argument of otherwise unaccusative verbs is rather rare and restricted to dynamic unaccusative verbs that potentially allow for agentive interpretations (in certain contexts). Stative unaccusative verbs like *matua* ‘be.tall’, which otherwise receive inalienable-genitive case, cannot occur with alienable genitive case at all (cf. Sorace and Shomura 2001 for similar restrictions on variable intransitivity in Japanese).

#### Inalienable genitive case on unergative subjects

Inalienable genitive case on unergative subjects is more common and primarily observed in two contexts (Mosel 1992): On the one hand, unergative subjects receive inalienable genitive case, when additional material intervenes in between the nominalizer *n* and VoiceP. This holds for unaccusative restructuring verbs like *fia* ‘be wanted’ (91) (cf. Collins 2017),^40^ the negative particle *lē* ‘be not’ (91) (cf. Mosel and Hovdhaugen 1992: 478, Mayer 1976: 344f), as well as aspectual particles like preverbal *toe* ‘again’ (92) and postverbal *so‘o* ‘frequently’ (93).

(91)

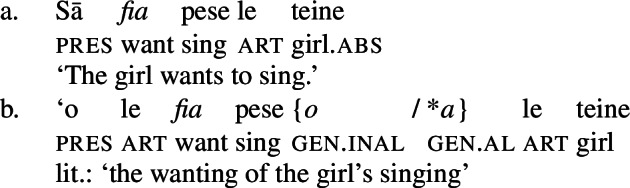

(92)

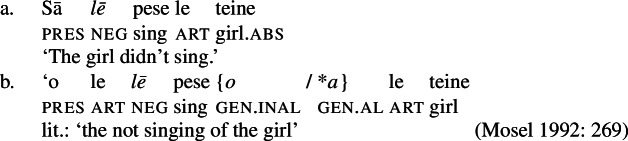

(93)
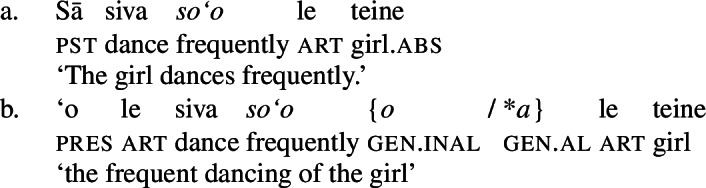

As the presence of intervening material, such as an AspP and a NegP breaks the selectional relationship between the nominalizer and the embedded verb, we expect that the unaccusativity constraint is lifted in such contexts, and VoiceP can introduce an external argument, as observed in the data. This also includes unaccusative restructuring verbs like *fia* ‘want’ (94), which independently satisfy the unaccusativity constraint.

(94)
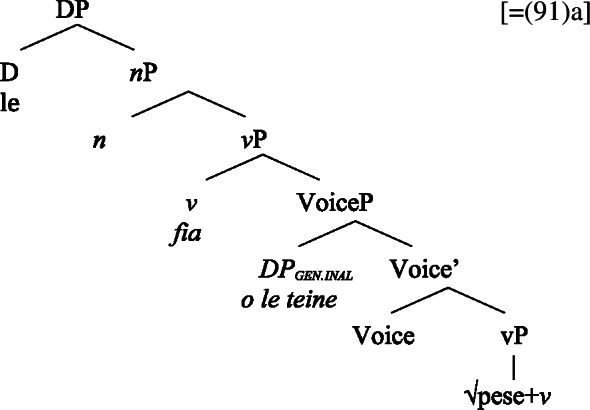

On the other hand, if unergative subjects receive inalienable genitive case in the absence of unaccusative restructuring verbs, Mosel and Hovdhaugen (1992) note that they are perceived as less agentive and less deliberate (95)b than their alienable-genitively marked counterparts (95)a (also Mosel 1992, cf. Moyse-Faurie 2000; Chung 1973 on the closely related Polynesian language Faka’uvea and Pukapukan, respectively). Notably, the genitive alternation also has an effect on the morphosyntactic properties of unergative nominalizations, as inalienable genitive case on subjects makes plural agreement available (95)b, which is otherwise ungrammatical on unergative subjects marked by alienable genitive case (see Sect. 2.4.2).^41^

(95)
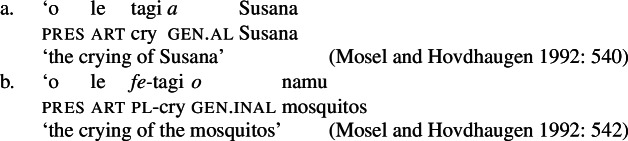

We take this variation as further indication that intransitive verbs do not necessarily split neatly into an unergative and an unaccusative class in Samoan, but allow their subjects to be merged either *v*P-internally or *v*P-externally: While high agentive intransitive subjects are located in Spec, VoiceP like transitive subjects, low agentive intransitive subjects are introduced within the *v*P (cf. Neu 2023, also Kouneli 2021; Tollan 2018; Massam 2009 for related intuitions). Consequently, low agentive subjects of otherwise unergative verbs do not violate the unaccusative requirement on nominalizations (96). In this context, unergative subjects therefore receive unmarked inalienable genitive case instead of alienable genitive case.^42^

(96)
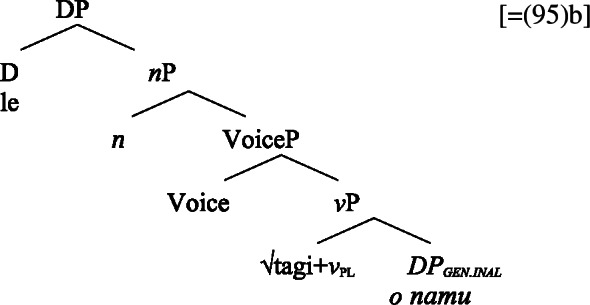

Samoan genitive case alternations therefore provide further crosslinguistic support against a categorial split of intransitive verbs into unaccusative and unergative types, as intransitive subjects can be variable, being introduced within or outside the *v*P, as previously observed for languages like Turkish or Italian (Neu 2023; Sorace 2000), though with interpretative consequences.

To summarize, we derived both the tripartite-inactive and the marked unergative alignment of Samon bare nominalizations from a language-specific combination of crosslinguistically established phenomena, i.e., split and variable intransitivity, prepositional ergativity, the unaccusative requirement on nominalizations, and the nonuniform nature of (subject) clitics. Our investigation therefore does not only provide additional support for each of those phenomena but also highlights the special status of unergative subjects in Samoan.

## On the source of ergativity

In closing, we briefly address further implications of our findings for the source of ergativity in Samoan and other Polynesian language based on the distribution of ergative morphology in nominalizations across languages. In particular, we demonstrate that inherent and dependent case approaches cannot readily explain the alignment types found in Samoan nominalizations. The prepositional analysis is also further supported by the distribution of the passive *by-*phrase in nominalizations of Polynesian languages with an accusative alignment, like Māori (Pearce 1997; Waite 1994). Contrasting the distribution of ergative marking in Samoan and Niuean nominalizations (Massam 2001), we then provide further evidence for a split between syntactically and morphologically ergative languages in Polynesian sensitive to the syntactic category of the ergative subject (Polinsky 2016).

### Against alternative analyses of ergativity in Samoan

While we have adopted a prepositional analysis of (syntactic) ergativity in Samoan (Polinsky 2016), there are previous accounts of the Samoan case system that treat ergative case as either an inherent case assigned by Voice (Tollan 2018), or as a dependent case, assigned to the highest DP in the clausal domain (Collins 2021). We now demonstrate that both approaches fail to account for the distribution of ergative and alienable genitive case in bare nominalizations, at least without further assumptions.

In dependent case approaches, ergative case is assigned to the structurally higher DP in the clausal domain, mirroring the assignment of accusative case in languages like English (Baker 2015; McFadden 2004; Marantz 1991*inter alia*). For Samoan, a version of dependent ergative case is adopted by Collins (2021) (see also Collins 2017, 2014). To account for syntactic ergativity, one may adopt a case discrimination account as proposed by Deal (2017) and Otsuka (2010), according to which ergative extraction is blocked, due to the sensitivity of A’-movement to morphological case (cf. Drummond 2023 on the Polynesian language Nukuoro).

(97)

In contrast, inherent case approaches treat ergative case as being inherently assigned to the DP argument in the specifier of a transitive Voice head (Legate 2008; Aldridge 2004; Woolford 1997*inter alia*). Adopting such an inherent case approach for Samoan, Tollan and Massam (2022) argue that syntactic ergativity follows from high absolutive case in the language, which requires the object to move across the ergative subject to the edge of the VP to get absolutive case from T (also Tollan 2018, cf. Clemens and Tollan 2021; Coon et al. 2014). As a result, the object argument freezes the ergative subject in its base-generated position for further (A’-)movement (see also Hopperdietzel 2022; Collins 2017 for independent motivation for object movement in Samoan).^43^

(98)

A crucial difference between the prepositional and both inherent and dependent case approaches to ergativity is the syntactic category of the ergative subject. While it is a PP-adjunct to VoiceP in the former, it is a DP argument situated in Spec, VoiceP in the latter. Inherent and dependent case approaches therefore predict the ergative subject to violate the unaccusative requirement on nominalizations and to pattern with unergative subjects, i.e., marked by alienable genitive case in Samoan nominalizations, contrary to the facts.^44^

(99)
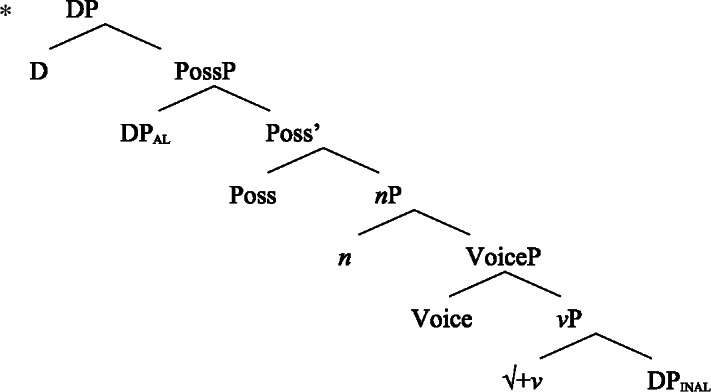

To save a DP-ergative analysis, one may assume that the unaccusative requirement does not hold for Samoan bare nominalizations (see Imanishi 2020 for a parametrized view on Mayan languages). However, without the unaccusative requirement, inherent alienable genitive case on unergative subjects is left unexplained, as unergative subjects would be expected to be introduced in Spec, VoiceP in the complement domain of *n*, receiving unmarked inalienable genitive case instead, again contrary to the facts.

(100)
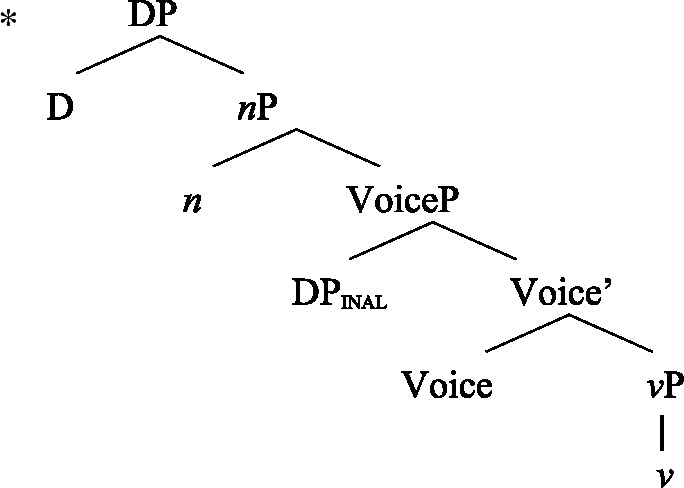

Therefore, inherent and dependent case approaches to Samoan ergativity face the same challenges as they are unable to account for (alienable genitively) marked unergative subjects in Samoan nominalizations without further assumptions.^45^ The prepositional account instead naturally derives the distribution of ergative and alienable genitive case nominalizations and links it to more general properties of the Samoan grammar, e.g., *ina* resumption for extracted subjects in the context of topicalized and clitisized subjects.

### Prepositional ergativity and passives: Māori

Crosslinguistic support for a prepositional analysis of Samoan ergativity comes from the related Eastern Polynesian language Māori (Harlow 2007). Unlike Samoan, Māori is described to exhibit an accusative-like alignment with intransitive and transitive subjects appearing in nominative case (marked by *a* for personal names) and objects in oblique case.

(101)
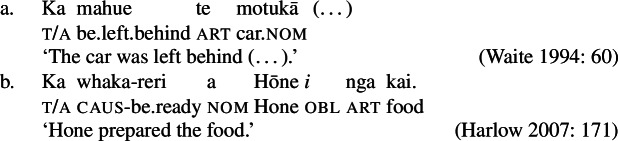

In addition, Māori has a passive construction marked by the passivizing suffix -C*ia* where the former subject is demoted to an oblique PP marked by *e* and the former object is promoted to the nominative subject. Note that the preposition *e* in the Māori passive is homophonous with the ergative marker *e* in Samoan.

(102)
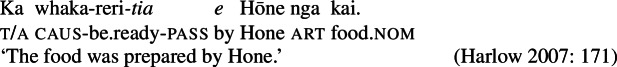

Similar to Samoan, Māori exhibits a type of bare nominalizations in which nominative case becomes unavailable (Pearce 1999; Waite 1994). In such nominalizations, unergative subjects are marked by inherent alienable (103)b and unaccuative subjects (103)a and objects by unmarked inalienable genitive case (see also Harlow 2007).

(103)
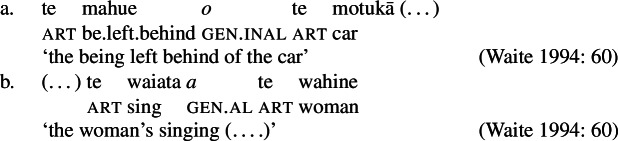

As expected from our investigation of Samoan nominalizations, transitive subjects do not receive inalienable genitive case. Instead, they can be realized as oblique PPs of nominalized passivized verbs, resembling passive *by-*phrases in Greek and ergative PPs in Samoan.^46^

(104)
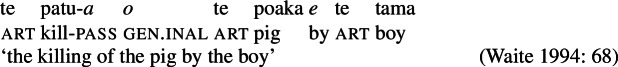

The passivization of transitive verbs together with the unavailability of inalienable genitive on transitive subjects in Māori nominalizations can again be explained by the unaccusative requirement, which prohibits the transitive subject to merge as a DP-argument in Spec, VoiceP (105). Unlike prepositional ergative languages like Samoan, accusative languages like Māori reflects this restriction morphologically with passive morphology on the verb.

(105)
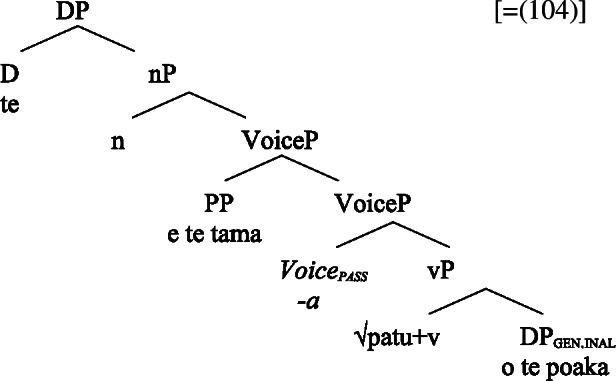

Crucially, in the Polynesian literature, passive *by-*phrases in Eastern Polynesian languages and ergative PPs in Western Polynesian languages have long been assumed to be diachronically related, providing a potential pathway for prepositional ergativity in Polynesian languages (cf. Chung 1978; Hohepa 1969).

### Morphological vs. syntactic ergativity: Niuean

In Sect. 4.1, we have argued that due to the unaccusativity restriction, ergative case is only expected to survive nominalization, if it is a prepositional case. Crucially, the difference between syntactic and mere morphological ergativity has been linked to the syntactic category of the ergative subject, i.e., PP- vs DP-ergatives (Polinsky 2016). For morphological ergative languages, we therefore predict the absence of ergative marking in nominalizations, as ergative DPs violate the unaccusative restriction. This prediction is borne out by Niuean.

In contrast to Samoan, Niuean (Polynesian) does not exhibit an extraction restriction on ergative subjects (Tollan and Massam 2022; Clemens and Tollan 2021; Polinsky 2016), as both absolutive and ergative arguments are topicalized without resumption (Massam 2020; Seiter 1980).^47^

(106)
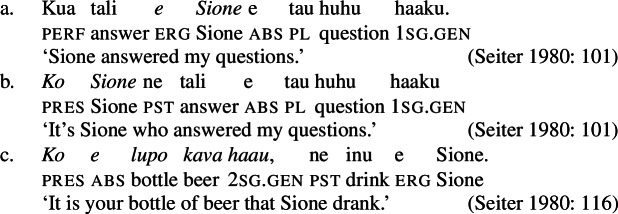

Consequently, Niuean ergativity can be classified as purely morphological. We therefore assume that ergative subjects are DP arguments merged in the specifier of transitive Voice with ergative case inherently or dependently assigned (Tollan and Massam 2022; Clemens and Tollan 2021; Massam 2020).^48^

(107)
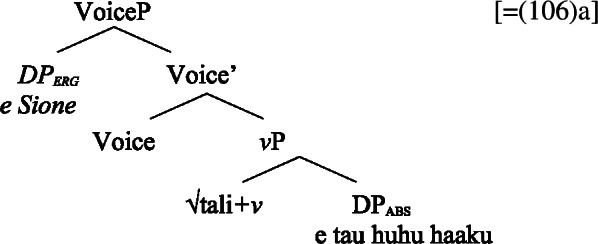

Unlike Māori however, Niuean lacks passive voice, i.e., it is unable to introduce the external argument as a PP. As is typical for many ergative languages (Dixon 1994), Niuean instead exhibits an antipassive construction to detransitivize transitive verbs, especially in imperfective contexts (Massam 2020, cf. Polinsky 2017 on the limited productivity of antipassives). In this construction, the former absolutive DP object is realized by an oblique locative PP with the subject in absolutive case.

(108)
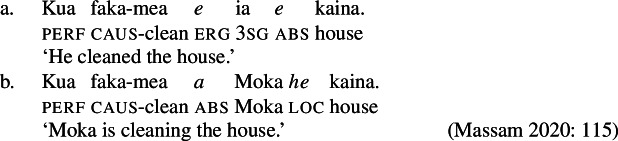

In contrast to passives in accusative languages like Greek or Māori, detransitivized verbs in Niuean are thus more similar to unergative than to unaccusative predicates, i.e., their Spec, VoiceP position is filled by a DP argument.

(109)
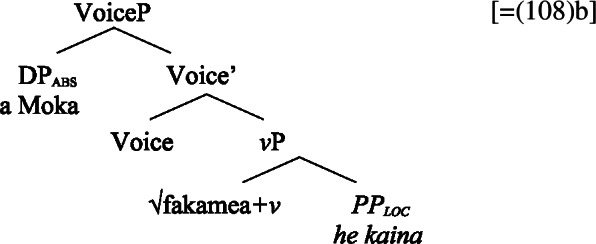

Like other Polynesian languages, Niuean exhibits a type of bare nominalizations, which has been proposed to exhibit selectional properties similar to their Samoan (and Greek) counterparts (Massam 2000, cf. Massam 2020 for different types of Niuean bare nominalizations).^49^ In contrast to Samoan however, ergative marking becomes unavailable in such nominalizations.^50^

(110)
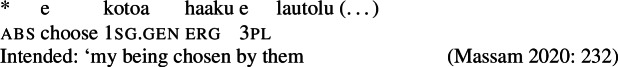

Instead, intransitive subjects (111)a and transitive subjects (111)b receive genitive case, whereas objects are introduced as PPs. Unlike Samoan and Māori, Niuean lacks split-alienability and assigns just a single genitive case, resulting in an accusative-type alignment in bare nominalizations (Massam 2000, but see Massam and Sperlich 2000 for a more complex picture).

(111)
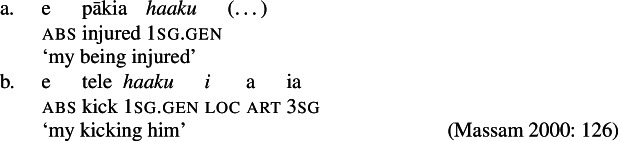

We can therefore attribute the variation between Samoan and Niuean to the interaction of the unaccusative requirement on nominalizations and the categorial status of transitive subjects. As ergative-marked transitive subjects are merged as DPs, they violate the unaccusative requirement. In the absence of a passive construction, transitive subjects must be merged as possessors in Spec, PossP in the nominal domain, where they receive unmarked genitive case (cf. Massam 2020). Due to the limited case licensing properties in Niuean nominals, we suggest that the internal argument must be introduced within a (locative) PP, akin to antipassive constructions above.

(112)
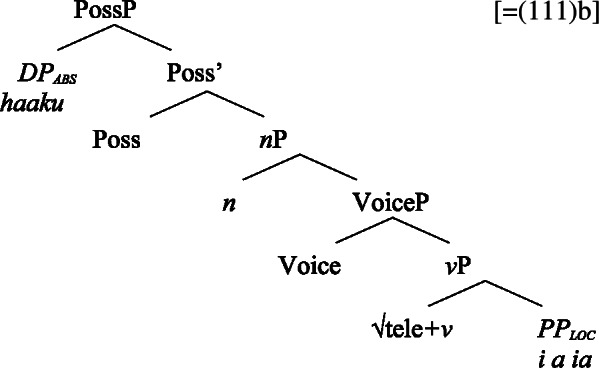

The absence of ergative case in Niuean nominalization therefore provides independent support for our hypothesis that only prepositional ergative case survives nominalization, whereas inherent/dependent ergative case does not, as it violates the unaccusative restriction on nominalizations. Consequently, nominalizations may be used as a diagnostic to identify the categorial status of ergative subjects, i.e., PP vs. DP, crosslinguistically.

## Conclusion

In this paper, we investigated the argument structure of Samoan bare nominalizations which show a crosslinguistically rare tripartite-inactive alignment of regular nouns that distinguishes unaccusative, unergative, and transitive subjects with an additional marked unergative alignment of subject clitics that singles out unergative subjects (Mosel 1992). By examining the morphosyntactic and semantic properties of bare nominalizations, we showed that these alignments follow naturally from a language-specific combination of independently established phenomena, including split intransitivity, split (in)alienability, prepositional syntactic ergativity, and a nonuniform status of subject clitics, for each of which our study thereby provides novel crosslinguistic support.

On the one hand, adopting a structural account of (in)alienablity, we identified alienable genitive case as an inherent case assigned to the DP in Spec, PossP, whereas inalienable genitive case represents the unmarked case in the nominal domain. The restriction of alienable genitive case to unergative subjects thus indicates that only unergative subjects are merged as possessors in the nominal domain, whereas all other arguments are introduced as event participants in the verbal domain. Classifying Samoan bare nominalizations as VoiceP-embedding *n*-based nominalizations, we explained the special status of unergative subjects by the unaccusative requirement on nominalization (Alexiadou 2001), according to which DPs are banned from Spec, VoiceP under *n*. Analyzing ergative marking as an inherent prepositional case in Samoan (cf. Polinsky 2016), transitive subjects satisfy this requirement, and ergative marking survives nominalization. In the absence of absolutive case, unaccusative subjects and objects receive unmarked inalienable genitive case, resulting in the tripartite-inactive case.

On the other hand, based on the prepositional nature of ergative subjects, we developed a nonuniform analysis of Samoan subject clitics (cf. Bleam 2000): Intransitive subject clitics are base-generated within a big-DP in their original *v*P-internal argument position, where they are assigned absolutive case before moving to T; transitive subject clitics are merged directly to T instead and require *ina-*resumption. In this position, transitive subject clitics receive unmarked absolutive case, resulting in a neutralized alignment. To satisfy the unaccusative requirement, only unergative subject clitics must be introduced in Spec, PossP, as transitive subject clitics can be merged directly to D. As a result, only unergative subjects are introduced as possessors of the nominalized event.

Our findings have broader theoretical implications: Firstly, the observation that the unaccusativity restriction on nominalizations holds in languages of typologically unrelated families, including Indo-European (Alexiadou 2001), Mayan (Imanishi 2020), and Polynesian, highlights the tight relation between nominalizations and unaccusativity. This restriction therefore calls for further examination across language types to gain a better understanding of its nature. Secondly, it supports a prepositional analysis of syntactic ergativity in Samoan, as analyses based on inherent or dependent ergative case cannot readily explain the case alignment of bare nominalizations. Thirdly, comparing the distribution of ergative case in nominalizations across ergative Polynesian languages, only PP-ergatives survive nominalizations (also Polinsky 2016 on Tongan), whereas DP-ergatives, as in the morphologically ergative language Niuean, violate the unaccusative requirement and are therefore ungrammatical in nominalizations (Massam 2020). However, the unaccusative requirement does not necessarily align with syntactic ergativity, since some syntactically ergative languages like Kaqchikel (Mayan) reject ergative case in nominalizations (Imanishi 2020, cf. Burukina 2023, 2021 for further discussion). This suggests that syntactic ergativity cannot be reduced to the categorial status of the ergative subject, PP vs. DP (*pace* Polinsky 2016) and therefore challenges a uniform crosslinguistic analysis (cf. Coon et al. 2014 for a related intuition).

## Footnotes

1 The glosses follow the Leipzig Glossing Rules, except: al = alienable, anitcaus = anticausative, cl = clitic, dim = diminutive, dir = directional, emph = emphatic, ergvz = ergativizer, inal = inalienable, incompl = incompletive, nspec = nonspecific, red = reduplication, rsmp = resumptive, spec = specific.

2 Our analysis is also compatible with a remnant movement account, as proposed by Collins (2017).

3 The investigation of Samoan *middle* verbs, i.e., psychological and communication verbs which trigger an absolutive/oblique alignment on their arguments (Chung 1978), is beyond the scope of this paper. While oblique arguments of middle verbs have been argued to share properties with (absolutive) objects (Tollan 2018), they pattern with true oblique arguments in others, e.g., both require the presence of the resumptive pronoun *ai* in dislocating contexts and survive in all kinds of nominalizations (see especially Cook 1988 for further arguments and critical discussion). In this study, we tentatively treat oblique objects of middle verbs as prepositional arguments, though additional research is required to discriminate their syntactic status.

4 As Samoan orthography does not reflect tonal marking, we do not mark absolutive case on the preceding syllable but on the lexical noun or the pronoun for the whole noun phrase for presentational convenience.

5 In colloquial speech, ergative case is sometimes dropped in Samoan (Mayer 2001; Ochs 1982; Duranti 1981). However, Yu and Stabler (2017: FN20) show that transitive subjects are not marked by tonal absolutive case but are truly unmarked. As our study focuses on formal Samoan, we do not expect any interferences.

6 In conversations, *ina* is often replaced by its reduced clitic form =*a*. Traditionally, (*in*)*a* is analyzed as a suffix, sometimes subsumed with the ergativizing suffix -C*ia* (Milner 1966; Cook 1988). Here, we follow Cook (1996) and Mosel (1985), who argue that *ina* and -C*ia* are distinct morphosyntactic entities, with the distribution of (*in*)*a* being sensitive to information structure, often with anaphoric function (cf. Cook 1994, 1978, Chung 1978).

7 A reviewer wonders whether *ina* could be the spell-out of traces of A’-movement of the ergative argument (Georgi and Amaechi 2023; van Urk 2018), implying that its presence does not serve as a diagnostic for syntactic ergativity. While a detailed analysis of *ina* resumption is beyond the scope of this paper, we briefly note that it does not exhibit morphosyntactic properties expected under a movement analysis (Hewett 2023): it lacks case identity and it does not license parasitic gaps/resumption (*pace* Ershova 2024, see Muāgututi’a 2018; Cook 1978 for interspeaker variation in the distribution of *ina*). Moreover, *ina* can double ergative subjects in postverbal position, i.e., in the absence of movement. Unexpected for a base-generation account, *ina* resumption is island sensitive (Ershova 2024). However, given that *ina* is void of any phi-features, it may to resemble bare resumptive pronouns in languages like Gaelic, which have been argued to require local binding via phasal Agree, contrasting with *phi-*matching pronouns (Adger 2011; Adger and Ramchand 2005).

8 Like *ina*, the resumptive clitic = *a* exhibits a corresponding demonstrative pro-form =*a* ‘this, that, the … in question’ which clitisizes to its preceding article, e.g., *l* = *a* (Mosel and Hovdhaugen 1992: 131f). Parallel to demonstratives, resumptive *ina*/=*a* is number-neutral and does not agree with its antecedent. We attribute the relation of demonstrative and anaphoric uses to future research.

9 Chung (1978) argues against apparent raising in Samoan partly based on examples from nineteenth-century texts where the ‘raised’ argument retains the case assigned in the embedded clause, which are, however, ungrammatical in contemporary Samoan. This is supported by our consultants who only accepted examples where the ‘raising’ verb assigns its own idiosyncratic case, e.g., ergative with *mafai* ‘be able to’ and absolutive with *masani* ‘be used to’. A second argument concerns the presence of *ina* in the embedded clause when the subject of a *mafai*-clause undergoes cliticization in the higher clause. While Chung takes *ina* to indicate extraction, we note that *ina* also occurs in nonmovement contexts (31), and thus its presence is not incompatible with an apparent raising analysis.

10 Given that we treat the ergative argument as a prepositional adjunct, two reviewers wonder whether our analysis overgenerates, since it seems to predict ergative PPs being compatible with unergative predicates in the absence of an internal argument. These structures, which would resemble impersonal passives in other languages, are unattested in Samoan. However, our analysis shares this issue with other inherent case analysis of ergativity, for which various solutions have been proposed. In particular, Tollan (2018) has argued for a split-Voice system in Samoan, according to which unergative subjects are introduced by a lower Voice head and transitive ones by a higher Voice head, based on their transitivity (cf. Polinsky 2016; Massam 2009 on Tongan and Niuean). Alternatively, Burukina and Polinsky (2025) and Coon et al. (2021) suggest that certain Voice heads in Mayan are equipped with a feature that requires checking by an internal argument, e.g., ergative assigning transitive Voice (see Hopperdietzel 2020 for a related proposal on Samoan where the relevant feature is inherited from T which may explain the difference between Voice in sentential and nominalization contexts). Finally, Williams (1987) explains the distribution of a special (ergative) marking of the external argument with a distinctness requirement in the presence of the internal argument. This distinctness approach is further pursued in Richards (2010) and Alexiadou (2001, to appear). In this paper, we remain agnostic about the exact implementation.

11 The classification of *a*/*o* genitives in terms of (in)alienability goes not without exceptions, as some nouns seem to determine the marking on the possessor. For example, while the body-part noun *vae* ‘hand’ triggers inalienable genitive case on the possessor, the noun *‘ava* ‘beard’ triggers alienable genitive case (Mosel and Hovdhaugen 1992: 285f). Such arbitrariness, which is also found in the context of Samoan kinship terms, has been noted for other languages and may not challenge the general intuition that *a*-marking corresponds to a more temporary, less-controlled type of possession (Heine 1997).

12 Outside of *ga-*nominalizations, alienable and inalienable genitive case cooccur rather rarely (cf. Clark 1981 on Polynesian). Note that Samoan also exhibits nominal compounds, which are primarily used in generic contexts, sometimes with idiosyncratic effects (Mosel and Hovdhaugen 1992: 239ff, 305ff).

13 As suggested by a reviewer, alienable case may alternatively be interpreted as a dependent case assigned inside the DP to the possessor which is in a c-command relationship with the possessum (cf. Baker 2015).

14 In addition to eventive readings, bare nominalizations of unergative verbs often have corresponding result nouns that are homophonous, such as *pese* ‘(the) song’ and *siva* ‘(the) dance’ (Milner 1966 on lexical categories in Samoan, cf. Hopperdietzel 2020; Don and van Lier 2013; Mosel and Hovdhaugen 1992: 77); though derived result nouns of unergative verbs also exist, e.g., *galue-ga* ‘(the) work’ (cf. Mosel and Hovdhaugen 1992: 195ff). Therefore, an expression like in (29)a/b can, in principle, also refer to ‘the song of the girl’ and ‘the laughter of the mother’, respectively. In this study, we controlled for the eventive reading, e.g., by the presence of cognate objects in Sect. 2.3.1, as we expect result nouns to exhibit distinct properties (as already indicated by Mosel 1992).

15 Mosel and Hovdhaugen (1992: 546) mention that less frequently, the object of transitive verbs, though not the subject of intransitive verbs, can be marked by absolutive case instead of inalienable genitive case, thus showing properties of an ABS=DEF system (see also Yu 2021; Collins 2014). Our consultants generally rejected absolutive case in bare nominalizations. Future research may investigate this interspeaker variation in more detail.

16 In colloquial speech, *ina* is commonly reduced to its clitisized form =*a* (Mosel and Hovdhaugen 1992).

17 Alternatively, *ina* doubling may be analyzed akin to some sort of passive formation under an analysis where the *by-*phrase is doubled by a (silent) pronoun (Sternefeld 1995, cf. also Baker et al. 1989). Accordingly, the ergative PP would be doubled by *ina* in Samoan (i) (cf. Nomoto 2022, 2016 on clitic doubling in passives in Malayo-Polynesian languages). As the exact status of *ina* doubling is not relevant for our purposes, we attribute a more detailed analysis to future research.

18 Note that overt category-changing morphology is rare in Samoan (Don and van Lier 2013, cf. Rijkhoff 2007).

19 As already noted by Cook (1991), verbal agreement is rarely used in contemporary Samoan with many speakers allowing plural agreement with the ergative argument as well.

20 Manner adverbs as a diagnostic for the presence of *v* is unavailable in Samoan, as there is no morphosyntactic way to distinguish between adverbs and adjectives, both appearing in their bare form (Mosel 2004).

21 The evidence for *n* must be indirect, as Samoan lacks grammatical gender, which has been located on *n* (e.g., Kramer 2016) and is used to diagnose the presence of (silent) *n* (Iordăchioaia 2020a).

22 In (55)b, verbal and nominal number cooccur. Here, the presence of verbal number indicates that the nominalized verb is not a result noun.

23 We follow Alexiadou et al. (2015), Legate (2014), and Bruening (2013) in analyzing *by-*phrases as prepositional adjuncts adjoined to VoiceP. On an alternative view, where *by-*phrases are treated as external (PP) arguments in Spec, VoiceP (e.g., Angelopoulos et al. 2020), the unaccusativity requirement in (58) would need to be revised to exclude DPs from Spec, VoiceP in nominalizations, but not PPs.

24 Bruening (2013) assumes that the unsaturated agent role is passed on to the nominal domain. While this may be the case for *n*-based nominalizations in some languages, nothing in our analysis hinges on this assumption, as long as Spec, VoiceP is not filled by an argument (but see Sect. 3.2.3 for interpretative reasons against this assumption for unergative subjects marked by alienable genitive case in Samoan nominalizations).

25 A reviewer notes that English equivalents of (61) may be grammatical, see (i). While we are not directly concerned with English nominalizations, we note that (ia) is a zero derived nominal, whose status as a complex event nominal is a matter of debate (Borer 2013; Grimshaw 1990). Iordăchioaia (2020b) shows that typically zero derived nominals from result verbs that combine with particles behave like complex event nominals. This does not hold for the example in (ia), which may be treated as a nonargument supporting nominal. (ib) is a gerund, which has been argued to contain VoiceP (see also FN26).

26 We note here that gerunds have been argued to lack an *n* head, thus they are not subject to this restriction. English seems to allow what is sometimes called transitive nominalizations, e.g., *the barbarian’s destruction of the city*. Following Grimshaw (1990), we analyze the prenominal possessor as an adjunct, introduced at the DP layer. As noted by Harley (2009: 324), the introduction of the possessor in the nominal domain also has semantic consequences, as they are not interpreted as agents but as possessors, which allows for various contextually available interpretations (see also Sect. 3.3). Such nominalizations are not allowed in Greek, a language in which Spec, DP has been shown to have A’-properties (Alexiadou et al. 2007 for discussion and references).

27 While several unergative verbs have cognate result nouns, such as *pese* ‘song’, *siva* ‘dance’, or *‘ata* ‘laughter’, some only exhibit derived forms such as *galue-ga* ‘work, job’, indicating that the distinction between result nouns and Voice-less complex event nominalizations is relevant in Samoan (see Alexiadou 2001 on Greek).

28 We note that subject-oriented modifiers like *ma le ita* ‘with anger’ are sensitive to the domain in which the subject is syntactically introduced. While unaccusative subjects that are introduced in the verbal domain can be targeted by such modifiers (i)a, unergative subjects that are introduced in the nominal domain cannot (i)b. As the syntax of *ma le X* constructions is unclear, especially since *ma* is also used in nominal and verbal coordination (Mosel and Hovdhaugen 1992: 301ff.), we attribute the source of this contrast to future research.

29 A reviewer wonders whether we also find nominalizations with a transitive subject marked by alienable genitive and the object by inalienable genitive, since both types of genitives can in principle cooccur. Chung (1978: 317) mentions that only one of her consultants accepted such an example. Our consultants all rejected examples like in (i). Note that for parallel examples in related Māori, significant interspeaker variation has been reported (Pearce 1997; Waite 1994), which is why we attribute the investigation of the nature of this restriction to future research.

30 There is some variation described for third-person subject clitics in the context of intransitive predicates (cf. Mosel and Hovdhaugen 1992; Chung 1978): Some speakers allow all kinds of intransitive subjects to be realized as clitics but others restrict them to agents (Cook 1991). Mosel (1987) notes that while unaccusative subject clitics are always ungrammatical, unergative subject clitics are grammatical in the plural and dual. We attribute this variation to future research. We also note that the restriction to subjects in Samoan may be a recent development, as clitic pronouns referring to absolutive object argument of transitive verbs is attested in Milner (1966). However, object clitics are judged ungrammatical in contemporary Samoan (Cook 1996; Mosel and Hovdhaugen 1992, but see Moyse-Faurie 1997 for object clitics in East Futunan).

31 While *ina* resumption is obligatory for left dislocated transitive subjects, it is only a strong tendency for clitic pronouns (Mosel 1985; Chung 1978; Cook 1978). Like *ina* doubling of in-situ ergative noun phrases, the distribution of *ina* may be determined by information structure, as it appears to highlight a topical discourse participant (Mosel 1985, but see Cook 1994 for an analysis based on low focus). Generally, the use of *ina* tends to be more frequent in formal registers (Mayer 2001; Duranti 1981), which is reflected by our consultants’ preference.

32 Note that third-person singular pronouns are homophonous with the demonstrative pronouns *nā* ‘that there’ and *ia* ‘this, that, the … in question (anaphoric)’ (Mosel and Hovdhaugen 1992: 131; cf. resumptive *ina*).

33 For some speakers, clitic doubling is generally degraded, though their judgments improve if the postverbal emphatic particle *fo‘i* is present. One reason may be that clitic doubling appears to have low status in language education, as indicated by one consultant, another that its discourse function was not met by our contexts.

34 The movement/base-generation contrast between absolutive and ergative clitics makes further predictions about their morphosyntactic behaviour. Given that subject clitics have only been superficially described in the literature, future research is needed to provide independent support for this split, which is beyond the scope of this paper.

35 A reviewer wonders whether resumption and clitic doubling as in sentences like (73)c and (74)b should be ruled by Principle B. (Principle C effects are not expected in Samoan, as subject clitic doubling is restricted to pronominal elements.) However, clitic doubling across languages has been shown to be insensitive to this kind of binding effects (Paparounas and Salzmann 2023; Baker and Kramer 2018, cf. Anagnostopoulou 2024, 2017 for a discussion). While the explanation of the absence of Principle B effects is therefore orthogonal to our analysis here, we note that the configuration in (75) may not satisfy the preconditions for binding, since the clitic as a D head is directly merged to the T head and thus not located in an A-position (such as Spec, TP, for example; see Baker and Kramer 2018 for a related intuition).

36 Genitive marking on plural and dual possessive clitics is optional (Mosel and Hovdhaugen 1992: 124ff). As mentioned above, similar instances of case drop have been described for ergative case in colloquial speech.

37 Unlike subject clitic doubling, possessive clitic doubling is rejected by our consultants. Therefore, possessive clitics may have been reanalyzed as weak pronouns, in the sense of Cardinaletti and Starke (1999). The exact status of possessive clitics is not directly relevant for our analysis. Like transitive subject clitics, transitive weak pronominal subjects would be externally merged in Spec, TP and resumed by resumptive *ina* in Spec, VoiceP. As our analysis is compatible with both clitic and weak pronouns, we attribute a more detailed investigation of the morphosyntactic variation of clitic pronouns to future research. As clitic doubling seems to be degraded even in the verbal domain, there might be an ongoing reanalysis of clitic as weak pronouns that affects possessive clitics first and spreads to subject clitics across Polynesian, resulting in decline of clitic doubling (cf. Macdonald 2014 for fully productive possessor doubling in Tongan, Kieviet 2017 for possessor doubling being restricted to second person in Rapa Nui, also Moyse-Faurie 1997 on the loss subject clitic doubling in Futunan).

38 The assignment of inalienable genitive case to the base-generated subject clitic in an athematic position further highlights the status of inalienable genitive case as the unmarked case in the DP. As a reviewer rightly notes, it would not be sufficient to say that inalienable genitive is unmarked case in the *n*P and alienable genitive the unmarked case in the DP, which may be the right analysis for split-genitive alignments in other languages.

39 A reviewer suggests that the internal argument position of *pa‘ū* ‘fall’ in its unergative form is occupied by a silent (arbitrary) *pro*. Since in its unergative use, *pa‘ū* patterns like other unergative verbs, we opt for a parallel treatment with an empty complement position. However, our analysis does not hinge on this assumption.

40 Unlike English *want*, Samoan *fia* ‘want’ is unable to introduce an additional ‘wanter’ argument. Instead, the embedded subject or object argument is inferred as the ‘wanter’ (cf. Mosel and Hovdhaugen 1992: 714). In the absence of more detailed studies, we assume that *fia* belongs to a group of unaccusative restructuring verbs, including *amata* ‘start, begin’ (cf. Wurmbrand 2001 on unaccusative restructuring verbs).

41 In addition, subject-oriented modification which is restricted to verbal arguments becomes available (cf. FN28).

42 Our analysis is compatible with different potential positions that have been proposed for the low agentive subject, e.g., the complement of *v* (Neu 2023) or Spec, *v*P (Tollan and Massam 2022).

43 High absolutive approaches to syntactic ergativity must assume an additional functional head F in the verbal domain above Voice that introduces a phase boundary (Clemens and Tollan 2021; Coon et al. 2014).

44 Samoan also exhibits *-ga* nominalizations that select for *v*P-complements (cf. Mosel and Hovdhaugen 1992: 194ff, 573ff, Mosel 1992; Don and van Lier 2013). In such contexts, Voice morphology like -C*ia* is unavailable, and transitive subjects are typically marked by alienable genitive case.

45 A reviewer suggests that inherent/dependent case approaches may obey the unaccusative requirement under the assumption that the ergative preposition is inserted as a spell-out of inherent/dependent ergative case on the transitive subject, similar to *of-*insertion in English. As rightly noted by the reviewer, P-insertion would need to take place before A’-movement, which is not in line with the standard assumption about inclusiveness and is not expected to change the syntactic status of the transitive subject from a DP-argument to a PP-adjunct.

46 Alternatively, the transitive subject can also be introduced as a possessor in the nominal domain, receiving alienable genitive case with the object being either an oblique argument (as in Samoan unergatives) or marked by inalienable genitive (see Pearce 1999; Waite 1994 for a more detailed overview).

47 In Niuean, ergative and absolutive case is *he* and *e* for proper and *e* and *a* for common nouns (Seiter 1980).

48 We remain agnostic about the locus of absolutive marking in Niuean, i.e., whether it is assigned low (ABS=DEF; Legate 2008, cf. Clemens and Tollan 2021; Massam 2020) or high (ABS=NOM; Heck 2025), as the relevant observation is that ergative case becomes unavailable in the nonfinite nominalizations.

49 Massam (2020) distinguishes ‘midlevel’ nominalizations, as discussed here, from ‘high’ nominalizations, which embed a finite clausal structure and show ergative and absolutive assignment in the context of transitive verbs (cf. Kornfilt and Whitman 2012). In her analysis, the locative argument is merged as an adjunct in the nominal domain. However, additional research is needed to fully establish the exact syntactic size of the verbal and the nominal domain in Niuean.

50 Seiter (1980) reports the example in (110) to be grammatical, though stylistically formal, for his consultants. However, Massam (2020) was not able to replicate this data in present-day Niuean.

## References

[CR1] Adger, David. 2011. Bare resumptives. In *Resumptive pronouns at the interfaces*, ed. Alain Rouveret, 343–366. Amsterdam: John Benjamins.

[CR2] Adger, David, and Gilian Ramchand. 2005. Merge and move: *Wh*-dependencies revisited. *Linguistic Inquiry* 36:161–193.

[CR3] Aldridge, Edith. 2004. *Ergativity and word order in Austronesian languages*, PhD thesis, Cornell University, Ithaca, NY.

[CR4] Alexiadou, Artemis. 1997. *Adverb placement: A case study in antisymmetric syntax*. Amsterdam: John Benjamins.

[CR5] Alexiadou, Artemis. 2001. *Functional structure in nominals: Nominalization and ergativity*. Amsterdam: John Benjamins.

[CR6] Alexiadou, Artemis. 2003. Some notes on the structure of alienable and inalienable possessors. In *From NP to DP*, eds. Martine Coene and Yves D’hulst. Vol. 2, 167–188. Amsterdam: John Benjamins.

[CR7] Alexiadou, Artemis. 2013. Nominal vs. verbal *-ing* constructions and the development of the English progressive. *English Linguistics Research* 2(2):126–140.

[CR8] Alexiadou, Artemis. 2017. Ergativity in nominalization. In *The Oxford handbook on ergativity*, eds. Jessica Coon, Diane Massam, and Lisa D. Travis, 355–372. Oxford: Oxford University Press.

[CR9] Alexiadou, Artemis. 2020. D vs. n nominalizations within and across languages. In *Nominalizations: 50 years on from Chomsky’s remarks*, eds. Artemis Alexiadou and Hagit Borer, 87–110. Oxford: OUP.

[CR10] Alexiadou, Artemis. All nominalizations big and small: Towards a typology of complex event nominals. In Proceedings of 60th Annual Meeting of the Chicago Linguistic Society. Vol. CLS60. To appear.

[CR11] Alexiadou, Artemis, and Hagit Borer, eds. 2020. *Nominalization: 50 years on from Chomsky’s remarks*, Oxford: Oxford University Press.

[CR12] Alexiadou, Artemis, Liliane Haegeman, and Melita Stavrou. 2007. *Noun phrase in the generative perspective*. Berlin: Mouton de Gruyter.

[CR13] Alexiadou, Artemis, Gianina Iordăchioaia, and Elena Soare. 2010. Number/aspect interactions in the syntax of nominalizations: A distributed morphology approach. *Journal of Linguistics* 46(3):537–574.

[CR14] Alexiadou, Artemis, Elena Anagnostopoulou, and Florian Schäfer. 2015. *External arguments in transitivity alternations: A layering approach*. Oxford: Oxford University Press.

[CR15] Anagnostopoulou, Elena. 2017. Clitic doubling, 2nd edn. In *The Wiley Blackwell companion to syntax*, eds. Martin Everaert and Henk C. Riemsdijk. Hoboken: Wiley.

[CR16] Anagnostopoulou, Elena. 2024. On the morpho-syntax of clitic doubling and object agreement: A view from Greek. Paper presented at Workshop: New Approaches to Clitics, University of Vienna.

[CR17] Angelopoulos, Nikos, Chris Collins, and Arhonto Terzi. 2020. Greek and English passives, and the role of *by*-phrases. *Glossa* 5(1):90.

[CR18] Armstrong, Grant. 2024. The roots and sructure of possessive noun classes. *Isogloss* 10(6):1–33.

[CR19] Baker, Mark C. 2015. *Case: Its principles and its parameters*. Cambridge: Cambridge University Press.

[CR20] Baker, Mark C., and Ruth Kramer. 2018. Doubled clitics are pronouns: Amharic objects (and beyond). *Natural Language & Linguistic Theory* 36:1035–1088.

[CR21] Baker, Mark C., Kyle Johnson, and Ian Roberts. 1989. Passive arguments raised. *Linguistic Inquiry* 20(2):219–251.

[CR22] Ball, Douglas. 2009. hili-clauses: Insights into Tongan nominalizations. In *Proceedings of the 16th annual meeting of the Austronesian Formal Linguistics Association (AFLA16)*, 1–15.

[CR23] Barker, Chris. 1995. *Possessive descriptions*. Stanford: CSLI Publications.

[CR24] Biggs, Bruce. 1969. *Let’s learn Maori*. Wellington: Reed.

[CR25] Bleam, Tonia M. 2000. *Leìsta Spanish and the syntax of clitic doubling*, PhD thesis, University of Delaware, Newark.

[CR26] Bobaljik, Jonathan, and Heidi Harley. 2017. Suppletion is local: Evidence from Hiaki. In *The structure of words at the interfaces*, eds. Heather Newell, Máire Noonan, Glyne Piggott, and Lisa D. Travis, 141–159. Oxford: Oxford University Press.

[CR27] Borer, Hagit. 2013. *Taking form: Structuring sense*, Vol. 3. Oxford: Oxford University Press.

[CR28] Borer, Hagit. 2014. Derived nominals and the domain of content. *Lingua* 141:71–96.

[CR29] Bruening, Benjamin. 2013. *By* phrases in passives and nominals. *Syntax* 16(1):1–41.

[CR30] Burukina, Irina. 2021. On the nature of arguments in event nominals. *Proceedings of the Linguistic Society of America* 6(1):996–1008.

[CR31] Burukina, Irina. 2023. Nominalized antipassive constructions in Kaqchikel. In *Proceedings of the workshop on the structure and constituency of languages of the America (WSCLA24)*, Vol. 24, 15–29.

[CR32] Burukina, Irina, and Maria Polinsky. 2025. The antipassive and verbal projections. Journal of Linguistics First View, 1–34.

[CR33] Burzio, Luigi. 1986. *Italian syntax: A government-binding approach*. Dordrecht: Springer.

[CR34] Cablitz, Gabriele H. 2000. Nominalization of verbal clauses in Marquesan (Oceanic, French Polynesia). In *Proceedings of the 7th annual meeting of the Austronesian Formal Linguistics Association (AFLA7)*, 1–14.

[CR35] Cardinaletti, Anna. 1998. On the deficient/strong opposition in possessive systems. In *Possessors, predicates, and movement in the determiner phrase*, eds. Artemis Alexiadou and Chris Wilder, 17–53. Amsterdam: Benjamins.

[CR36] Cardinaletti, Anna. 2019. Cliticization as extraction: The big DP hypothesis revisited. *Revista da Associação Portuguesa de Linguistica* 5:1–16.

[CR37] Cardinaletti, Anna, and Michal Starke. 1999. The typology of structural deficiency: A case study of three classes of pronouns. In *Eurotyp, Vol 5., Clitics in the Languages of Europe, Part 1*, ed. Henk van Riemsdijk, 145–234. Berlin: De Gruyter Mouton.

[CR38] Chomsky, Noam. 1981. *Lectures on government and binding*. Dordrecht: Foris.

[CR39] Chung, Sandra. 1973. The syntax of nominalizations in Polynesian. *Oceanic Linguistics* 12(1/2):641–686.

[CR40] Chung, Sandra. 1978. *Case marking and grammatical relations in Polynesian*. Austin: University of Texas Press.

[CR41] Cinque, Guglielmo. 1999. *Adverbs and functional heads: A cross-linguistic perspective*. Oxford: Oxford University Press on Demand.

[CR42] Clark, Ross. 1981. Inside and outside Polynesian nominalizations. In *Studies in Pacific languages and cultures in honour of Bruce Biggs*, eds. Jim Hollyman and Andrew Pawley. Auckland: Linguistic Society of New Zealand, 65–81.

[CR43] Clark, Ross. 2000. Possessive markers in Polynesian languages. *STUF – Language Typology and Universals* 53(3/4) 258–268.

[CR44] Clemens, Lauren E. 2014. *Prosodic noun incorporation and verb initial syntax*, PhD thesis, Harvard University, Cambridge, MA.

[CR45] Clemens, Lauren E., and Rebecca Tollan. 2021. Syntactic ergativity and word order in Tongic languages. In *Polynesian syntax and its interfaces*, eds. Lauren E. Clemens and Diane Massam, 89–112. Oxford: Oxford University Press.

[CR46] Collins, James N. 2014. The distribution of unmarked cases in Samoan. In *Argument realisations and related constructions in Austronesian languages*, eds. I. Wayan Arka and N. L. K. Mas Indrawati, 93–110. Canberra: Pacific Linguistics.

[CR47] Collins, James N. 2017. Samoan predicate initial word order and object positions. *Natural Language & Linguistic Theory* 35(1):1–59.

[CR48] Collins, James N. 2021. Mapping meanings to argument structure: The semantics of Samoan case. In *Polynesian syntax and its interfaces*, eds. Diane Massam and Lauren E. Clemens, 36–60. Oxford: Oxford University Press.

[CR49] Comrie, Bernard. 2013. Alignment of case marking of full noun phrases. In *The world atlas of language structures online*, eds. Matthew S. Dryer and Martin Haspelmath. Leipzig: Max Planck Institute for Evolutionary Anthropology.

[CR50] Cook, Kenneth W. 1978. The mysterious Samoan transitive suffix. In *Proceedings of the 4th annual meeting of the Berkeley Linguistics Society (BLS4)*, 53–66.

[CR51] Cook, Kenneth W. 1988. *A cognitive analysis of grammatical relations, case, and transitivity in Samoan*, PhD thesis, University of California, San Diego.

[CR52] Cook, Kenneth W. 1991. The search for subject in Samoan. In *Currents in Pacific linguistics: Papers on Austronesian languages and ethnolinguistics in honour of George W. Grace*, ed. Robert Blust. Canberra: Pacific Linguistics.

[CR53] Cook, Kenneth W. 1994. The empathy hierarchy and Samoan clitic pronouns. *Cognitive Linguistics* 5(1):57–75.

[CR54] Cook, Kenneth W. 1996. The cia suffix as a passive marker in Samoan. *Oceanic Linguistics* 35(1):57–76.

[CR55] Coon, Jessica. 2013. *Aspects of split-ergativity*. Oxford: Oxford University Press.

[CR56] Coon, Jessica, Pedro Mateo Pedro, and Omer Preminger. 2014. The role of case in A-bar extraction asymmetries: Evidence from Mayan. *Linguistic Variation* 14(2):179–242.

[CR57] Coon, Jessica, Nico Baier, and Theodore Levin. 2021. Mayan agent focus and the ergative extraction constraint: Facts and fictions revisited. *Language* 97(2):269–332.

[CR58] Cuervo, María C. 2003. *Datives at large*, PhD thesis, Massachusetts Institute of Technology, Cambridge, MA.

[CR59] de Miguel, Elena. 1996. Nominal infinitives in Spanish: An aspectual constraint. *Canadian Journal of Linguistics* 41(1):29–54.

[CR60] Deal, Amy Rose 2017. Syntactic ergativity as case discrimination. In *Proceedings of the 34th meeting of the West coast conference on formal linguistics (WCCFL34)*, 141–150.

[CR61] Dixon, Robert M. W. 1994. *Ergativity*. Cambridge: Cambridge University Press.

[CR62] Don, Jan, and Eva van Lier. 2013. Derivation and categorization in flexible and differentiated languages. In *Flexible word classes: Typological studies of underspecified parts-of-speech*, eds. Jan Rijkhoff and Eva van Lier, 56–88. Oxford: Oxford University Press.

[CR63] Drummond, Emily. 2023. *Clause structure and ergativity in Nukuoro*, PhD thesis, University of California, Berkeley.

[CR64] Duranti, Alessandro. 1981. The Samoan fono: A sociolinguistic study. Canberra: Linguistic Circle of Canberra.

[CR65] Ershova, Ksenia. 2024. Φ-feature mismatches in Samoan resumptives as postsyntactic impoverishment. In *Proceedings of the 59th Annual Meeting of the Chicago Linguistic Society (CLS59)*, 87–102.

[CR66] Georgi, Doreen, and Mary Amaechi. 2023. Resumption in Igbo: Two types of resumptives, complex phi-mismatches, and dynamic deletion domains. *Natural Language & Linguistic Theory* 41:961–1028.

[CR67] Grewendorf, Günther. 2008. The clausal left periphery: Clitic left dislocation in Italian and left dislocation in German. In *Dislocated elements in discourse: Syntactic, semantic, and pragmatic perspectives*, eds. Benjamin Shaer, Philippa Cook, Werner Frey, and Claudia Maienborn, 49–94. London: Routledge.

[CR68] Grimshaw, Jane. 1990. *Argument structure*. Cambridge: MIT Press.

[CR69] Haji-Abdolhosseini, Mohammad, Diane Massam, and Kenji Oda. 2002. Number and events: Verbal reduplication in Niuean. *Oceanic Linguistics* 41(2):475–492.

[CR70] Harley, Heidi. 2009. The morphology of nominalizations and the syntax of vP. In *Quantification, definiteness and nominalization*, eds. Monika Rathert and Anastasia Giannadikou, 320–342. Oxford: Oxford University Press.

[CR71] Harlow, Ray. 2007. *Māori: A linguistic introduction*. Cambridge: Cambridge University Press.

[CR72] Haugen, Jason. 2011. Reduplication in distributed morphology. In *Coyote papers: Working papers in linguistics, linguistic theory at the University of Arizona*, Vol. 18, 1–27.

[CR73] Heck, Fabian. 2025. On split-absolutive. In *Strict cycling: A festschrift for Gereon Müller*, eds. Silke Fischer, et al.. 179–202. Leipzig: Institut für Linguistik.

[CR74] Heine, Bernd. 1997. *Possession: Cognitive sources, forces, and gramaticalization*. Cambridge: Cambridge University Press.

[CR75] Hewett, Matthew R. 2023. *Types of resumptive A’-dependencies*, PhD thesis, University of Chicago.

[CR76] Hohaus, Vera, and Anna Howell. 2015. Alternative semantics for focus and questions: Evidence from Samoan. In *Proceedings of the 21st meeting of the Austronesian formal linguistics association (AFLA21)*, 69–86.

[CR77] Hohepa, Partick W. 1969. The accusative-to-ergative drift in Polynesian languages. *Journal of the Polynesian Society* 78:295–329.

[CR78] Holmer, Arthur. 1999. An active analysis of Basque ergativity. *Fontes Linguae Vasconum* 31:189–225.

[CR79] Hooper, Robin. 1996. Type and instance nominalizations Tokelauan. In *Proceedings of the first international conference on Oceanic linguistics (COOL1)*, 223–241.

[CR80] Hooper, Robin. 2000. Possessive markers in Tokelauan. *STUF – Language Typology and Universals* 53(3/4) 293–307.

[CR81] Hopperdietzel, Jens. 2020. *Resultatives: A view from Oceanic verb serialization*, PhD thesis, Humboldt University of Berlin.

[CR82] Hopperdietzel, Jens. 2021. Causative morphology as Voice-driven allomorphy: The case of Samoan fa’a-causatives. In *Polynesian syntax and its interfaces*, eds. Diane Massam and Lauren E. Clemens. Oxford: Oxford University Press.

[CR83] Hopperdietzel, Jens. 2022. Talmy’s typology in serializing languages: Variations on a VP. *Glossa* 7(1).

[CR84] Imanishi, Yusuke. 2014. *Default ergative*, PhD thesis, Massachusetts Institute of Technology, Cambridge, MA.

[CR85] Imanishi, Yusuke. 2020. Parametrizing split ergativity in Mayan. *Natural Language & Linguistic Theory* 38(1):151–200.

[CR86] Iordăchioaia, Gianina. 2014. The interaction between *n*P and DP in nominalizations. *Proceedings of NELS* 43(ELS43):179–190.

[CR87] Iordăchioaia, Gianina. 2020a. D and N are different nominalizers. *Glossa* 5(1):53.

[CR88] Iordăchioaia, Gianina. 2020b. Event structure and argument realization in English zero-derived nominals with particles. *Nordlyd* 44(1):35–51.

[CR89] Kahnemuyipour, Arsalan, and Diane Massam. 2006. Patterns of phrasal movement: The Niuean DP. In *Clause structure and adjuncts in Austronesian languages*, eds. Hans-Martin Gärtner, Paul Law, and Joachim Sabel, 125–150. Berlin: De Gruyter Mouton.

[CR90] Kieviet, Paulus. 2017. *A grammar of Rapa Nui*. Berlin: Language Science Press.

[CR91] Koopman, Hilda. 2012. Samoan ergativity as double passivization. In *Functional heads: The cartography of syntactic structures*, eds. Laura Brugé, et al.. Vol. 7, 168–180. Oxford: Oxford University Press.

[CR92] Koptjevskaja-Tamm. 1993. *Nominalizations*. Abingdon: Routledge.

[CR93] Kornfilt, Jaklin, and John Whitman. 2012. Genitive subjects in TP nominalizations. *Proceedings of JeNom* 4:39–72.

[CR94] Kouneli, Maria. 2021. Low (in)transitivity: Evidence from Kipsigis. In *Proceedings of the 51st Meeting of the North East Linguistic Society (NELS51)*, 53–66.

[CR95] Kramer, Ruth. 2016. The location of gender features in the syntax. *Language and Linguistics Compass* 10(11):661–677.

[CR96] Kratzer, Angelika. 1996. Severing the external argument from its verb. In *Phrase structure and the lexicon*, eds. Johan Rooryck and Laurie Zaring, 109–137. Dordrecht: Kluwer.

[CR97] Legate, Julie A. 2008. Morphological and abstract case. *Linguistic Inquiry* 39(1):55–101.

[CR98] Legate, Julie A. 2014. *Voice and v: Lessons from Acehnese*. Cambridge: MIT Press.

[CR99] Lewis, M. Paul, Gary F. Simons, and Charles D. Fenning. 2015. *Ethnologue: Languages of the world*, 16th edn. Dallas: SIL International.

[CR100] Macdonald, Catherine M. 2014. *Functional projections and non-local relations in Tongan nominal phrases*, PhD thesis, University of Toronto.

[CR101] Marantz, Alec. 1991. Case and licensing. In *Proceedings of the 8th Eastern States Conference on Linguistics (ESCOL8)*, 234–253.

[CR102] Massam, Diane. 2000. Niuean nominalisation. In *Proceedings of AFLA7*, 121–133.

[CR103] Massam, Diane. 2001. Pseudo noun incorporation in Niuean. *Natural Language & Linguistic Theory* 19(1):153–197.

[CR104] Massam, Diane. 2009. The structure of (un)ergatives. In *Proceedings of the 16th meeting of the Austronesian formal linguistics association (AFLA16)*, 125–135.

[CR105] Massam, Diane. 2020. *Niuean: Predicates and arguments in an isolating language*. Oxford: Oxford University Press.

[CR106] Massam, Diane, and Wolfgang B. Sperlich. 2000. Possession in Niuean. *STUF – Language Typology and Universals* 53(3/4) 281–292.

[CR107] Mayer, John. 1976. *Samoan Language: A manual for the study and teaching of the Samoan language as taught by Peace Corps/Western Samoa*. Washington: Peace Corps.

[CR108] Mayer, John. 2001. *Code-switching in Samoan: t-style and k-style*, PhD thesis, University of Hawai’i at Manoa, Honolulu.

[CR109] McFadden, Thomas. 2004. *The position of morphological case in the derivation: A study on the syntax-morphology interface*, PhD thesis, University of Pennsylvania, Philadelphia.

[CR110] Medeiros, David. 2013. Hawai’ian VP-remnant movement: A cyclic linearization approach. *Lingua* 127:72–97.

[CR111] Middleton, John. 2021. Revisiting the clause periphery in Polynesian languages. *Glossa* 6(1):87.

[CR112] Milner, George B. 1966. *Samoan dictionary*. London: Oxford University Press.

[CR113] Mosel, Ulrike. 1985. *Ergativity in Samoan*. Cologne: Department of Linguistics.

[CR114] Mosel, Ulrike. 1987. Subject in Samoan. In *A world of language: Papers presented to Professor S. A. Wurm on his 65th birthday*, eds. Donald C. Laycock and Werner Winter, 455–479. Canberra: Pacific Linguistics.

[CR115] Mosel, Ulrike. 1991. Transitivity and reflexivity in Samoan. *Australian Journal of Linguistics* 11(2):175–194.

[CR116] Mosel, Ulrike. 1992. On nominalisations in Samoan. In *The language game: Papers in the memory of Donald C. Laycock*, eds. Tom Dutton, Malcolm Ross, and Darrell Tyron, 263–281. Canberra: Pacific Linguistics.

[CR117] Mosel, Ulrike. 2004. Complex predicates and juxtapositional constructions in Samoan. In *Complex predicates in Oceanic languages: Studies in the dynamics of binding and boundness*, eds. Isabelle Bril and Françoise Ozanne-Rivierre, 263–296. Berlin: Mouton de Gruyter.

[CR118] Mosel, Ulrike, and Even Hovdhaugen. 1992. *Samoan reference grammar*. Oslo: Scandinavian University Press.

[CR119] Moyse-Faurie, Claire. 1997. Syntactic and pragmatic functions of pronominal arguments in some western Polynesian languages. *Oceanic Linguistics* 36:6–28.

[CR120] Moyse-Faurie, Claire. 2000. Possessive markers in East Uvean (Faka’uvea). *STUF – Language Typology and Universals* 53(3/4):319–332.

[CR121] Moyse-Faurie, Claire. 2016. Referential markers in Oceanic nominalized constructions. In *Finiteness and nominalization*, eds. Claudine Chamoreau and Zarina Estrada-Fernandez, 171–204. Amsterdam: John Benjamins.

[CR122] Muāgututi’a, Grant. 2018. *Recovering ergativity in heritage Samoan*, PhD thesis, University of Hawai’I at Manoa, Honolulu.

[CR123] Myler, Neil. 2016. *Building and interpreting possession sentences*. Cambridge: MIT Press.

[CR124] Neu, Eva. 2023. Against a low subject analysis of causatives of unergatives. *Proceedings of the Linguistics Society of America* 8(1):5527.

[CR125] Nomoto, Hiroki. 2016. Passives and clitic doubling. In *The proceedings of the 23rd Austronesian formal linguistics association (AFLA23)*, 219–236.

[CR126] Nomoto, Hiroki. 2022. Ergative extraction and the emergence of the active voice in Sumbawa. In *The proceedings of the 28th meeting of the Austronesian formal linguistics association (AFLA28)*, 95–111.

[CR127] Ochs, Eleonor. 1982. Ergativity and word order in Samoan child language. *Language* 58(3):646–671.

[CR128] Otsuka, Yuko. 2005. Two derivations of VSO: A comparative study of Niuean and Tongan. In *Verb first: On the syntax of verb-initial languages*, eds. Andrew Carnie, Heidi Harley, and Sheila Ann Dooley, 65–90. Amsterdam: John Benjamins.

[CR129] Otsuka, Yuko. 2010. DP ellipsis in Tongan: Is syntactic ergativity real? *Natural Language & Linguistic Theory* 28(2):315–342.

[CR130] Otsuka, Yuko. 2011. Neither accusative nor ergative: An alternative analysis of case in eastern Polynesian. In *Topics in Oceanic morphosyntax*, eds. Claire Moyse-Faurie and Joachim Sabel, 289–318. Berlin: De Gruyter Mouton.

[CR131] Paparounas, Lefteris, and Martin Salzmann. 2023. First conjunct clitic doubling, the Person Case Constraint, and first conjunct agreement: Insights from Modern Greek. *Glossa* 8.

[CR132] Pearce, Elizabeth. 1997. Genitive case in the Maori DP. *Wellington Working Papers in Linguistics* 9:31–56.

[CR133] Pearce, Elizabeth. 1999. Topic and focus in a head-initial language: Maori. In *Proceedings of the sixth meeting of the Austronesian formal linguistics association (AFLA6)*, 249–263.

[CR134] Pearce, Elizabeth. 2005. Iterative phrasal movement and the Maori DP. Ms. Victoria University of Wellington.

[CR135] Perlmutter, David. 1978. Impersonal passives and the unaccusative hypothesis. *Papers from the annual meeting the Berkeley Lingusitic Society* 4:157–189.

[CR136] Polinsky, Maria. 2016. *Deconstructing ergativity: Two types of ergative languages and their features*. Oxford: Oxford University Press.

[CR137] Polinsky, Maria. 2017. Antipassive. In *The Oxford handbook of ergativity*, eds. Jessica Coon, Diane Massam, and Lisa D. Travis, 308–331. Oxford: Oxford University Press.

[CR138] Potsdam, Eric, and Maria Polinsky. 2011. Questions and word in Polynesian. In *Topics in Oceanic morphosyntax*, eds. Claire Moyse-Faurie and Joachim Sabel, 83–109. Berlin: de Gruyter.

[CR139] Richards, Norvin. 2010. *Uttering trees*. Cambridge: MIT Press.

[CR140] Rijkhoff, Jan. 2007. *Language and Linguistics Compass* 1(6):709–726.

[CR141] Salanova, Andrés P. 2007. *Nominalizations and aspect*, PhD thesis, Massachusetts Institute of Technology, Cambridge, MA.

[CR142] Seiter, William J. 1980. *Studies in Niuean syntax*, New York: Garland.

[CR143] Šereikaitė, Milena. 2023. *Voice and case properties of complex event nominalizations: A voice-bundling approach. Ms*. Princeton: Princeton University.

[CR144] Shibatani, Masayoshi. 2006. On the conceptual framework for voice phenomena. *Linguistics* 44(2):217–269.

[CR145] Smirnova, Anastasia, and Ray Jackendoff. 2017. Case assignment and argument realization in nominals. *Language* 93(4):877–911.

[CR146] Sorace, Antonella. 2000. Gradience at the lexicon-syntax interface: Evidence from auxiliary selection and implications for unaccusativity. In *The unaccusativity puzzle*, eds. Artemis Alexiadou, Elena Anagnostopoulou, and Martin Everaert, 243–268. Oxford: Oxford University Press.

[CR147] Sorace, Antonella, and Yoko Shomura. 2001. Lexical constraints on the acquisition of split intransitivity. *Studies in Second Language Acquisition* 23:247–278.

[CR148] Sportiche, Dominique. 1996. Clitic constructions. In *Phrase structure and the lexicon*, eds. Johan Rooryck and Laurie Zaring, 213–276. Dordrecht: Kluwer.

[CR149] Sternefeld, Wolfgang. 1995. Voice phrases and their specifiers. *FAS Papers in Linguistics* 3:48–85.

[CR150] Szabolsci, Anna. 1994. The noun phrase. In *The syntactic structure of Hungarian, 179-214*, eds. Ferenc Kiefer and Katalin É. Kiss. San Diego: Academic Press.

[CR151] Thornton, Abigail. 2019. Agreeing in number: Verbal plural suppletion and reduplication. *The Linguistic Review* 36(3):531–552.

[CR152] Tollan, Rebecca. 2018. Unergatives are different: Two types of transitivity in Samoan. *Glossa* 3(1), 35. 10.5334/gjgl.223.

[CR153] Tollan, Rebecca, and Diane Massam. 2022. Licensing unergative objects in ergative languages: The view from Polynesian. *Syntax* 25(2) 242–275.

[CR154] Toosarvandani, Maziar. 2016. Vocabulary insertion and locality: Verbal suppletion in Northern Paiute. In *Proceedings of the 46th annual meeting of North East linguistic society (NELS46/3)*, 247–257.

[CR155] Tyler, Matthew. 2021. Two kinds of external possession in Mississippi Choctaw. *Syntax* 24(1):78–122.

[CR156] Uriagereka, Juan. 1995. Aspects of the syntax of clitic placement in western romance. *Linguistic Inquiry* 26(1):79–123.

[CR157] van Lier, Eva, and Marlou van Rijn. 2013. Argument coding in nominalizations of central-eastern Oceanic languages. *Lingue E Linguaggio* 12(2):279–305.

[CR158] van Urk, Coppe. 2018. Pronoun copying in Dinka Bor and the copy theory of movement. *Natural Language & Linguistic Theory* 36(3):937–990.

[CR159] van Urk, Coppe. 2022. Constraining predicate fronting. *Linguistic Inquiry* early access 1–47.

[CR160] Waite, Jeffrey. 1994. Determiner phrases in Maori. *Te Reo* 37:55–70.

[CR161] Williams, Edwin. 1987. English as an ergative language: The theta-structure of derived nouns. In *Proceedings of the 23rd annual regional meeting of the Chicago Linguistics Society (CLS23/1)*, Vol. 1, 366–375.

[CR162] Wilson, William H. 1982. *Proto-Polynesian possessive marking*. Canberra: Pacific Linguistics.

[CR163] Woolford, Ellen. 1997. Four-way case systems: Ergative, nominative, objective and accusative. *Natural Language & Linguistic Theory* 15(1):181–227.

[CR164] Wurmbrand, Susi. 2001. *Infinitives: Restructuring and clause structure*. Berlin: Mouton de Gruyter.

[CR165] Yu, Kristine M. 2021. Tonal marking of absolutive case in Samoan. *Natural Language & Linguistic Theory* 39:291–365.

[CR166] Yu, Kristine M., and Edward Stabler. 2017. (In)variability in the Samoan syntax/prosody interface and consequences for syntactic parsing. *Laboratory Phonology* 8(1):25. 10.5334/labphon.113.

[CR167] Zuraw, Kie, Kristine M. Yu, and Robyn Orfitelli. 2014. The word-level prosody of Samoan. *Phonology* 31(2). 10.1017/S095267571400013X.

